# A plastic relationship between vinculin-mediated tension and adhesion complex area defines adhesion size and lifetime

**DOI:** 10.1038/ncomms8524

**Published:** 2015-06-25

**Authors:** Pablo Hernández-Varas, Ulrich Berge, John G. Lock, Staffan Strömblad

**Affiliations:** 1 Karolinska Institutet, Department of Biosciences and Nutrition, Novum, Hälsovägen 7, Huddinge SE 141 83, Sweden

## Abstract

Cell-matrix adhesions are central mediators of mechanotransduction, yet the interplay between force and adhesion regulation remains unclear. Here we use live cell imaging to map time-dependent cross-correlations between vinculin-mediated tension and adhesion complex area, revealing a plastic, context-dependent relationship. Interestingly, while an expected positive cross-correlation dominated in mid-sized adhesions, small and large adhesions display negative cross-correlation. Furthermore, although large changes in adhesion complex area follow vinculin-mediated tension alterations, small increases in area precede vinculin-mediated tension dynamics. Modelling based on this mapping of the vinculin-mediated tension-adhesion complex area relationship confirms its biological validity, and indicates that this relationship explains adhesion size and lifetime limits, keeping adhesions focal and transient. We also identify a subpopulation of steady-state adhesions whose size and vinculin-mediated tension become stabilized, and whose disassembly may be selectively microtubule-mediated. In conclusion, we define a plastic relationship between vinculin-mediated tension and adhesion complex area that controls fundamental cell-matrix adhesion properties.

## Abstract (web summary)

Cell-matrix adhesions may increase or decrease in size in response to tension; however, the factors determining which of these responses predominates remain unclear. Hernández-Varas *et al*. quantify the plastic relationship between adhesion size and tension and use modelling to explain this behaviour.

## Graphical abstract

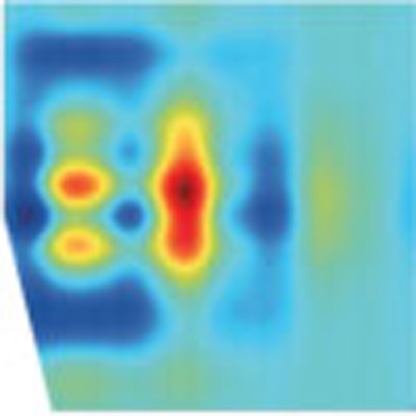

Cell adhesion to the extracellular matrix (ECM) governs a wide range of cellular processes, including differentiation, survival, proliferation and migration. Deregulation of cell-ECM adhesion can cause a range of pathologies, including aberrant cell migration enabling cancer cell metastasis^1^. Cell-ECM adhesion transmits mechanical forces bi-directionally between the cellular microenvironment and the cytoskeleton, with broad implications in stem cell differentiation and cancer progression^2^,^3^,^4^. However, the regulatory interplay between mechanical tension and cell-matrix adhesion remains unclear.

Cell-ECM adhesion is principally mediated by integrins, which bind ECM ligands via their ecto-domains, and a large spectrum of intracellular signalling and adaptor proteins via their cytoplasmic tails. The resulting macromolecular assemblages, known as cell-matrix adhesion complexes (CMACs), physically link the actin cytoskeleton with the ECM, acting as chemical and mechanical signalling hubs^5^,^6^, representing foci for bi-directional mechanotransduction^7^. However, only a few specific adhesion complex proteins are known to play key roles in mechanotransduction^8^. Such load-bearing proteins, localized within the CMAC-cytoskeleton linkage, undergo conformational changes in response to tensile forces^9^. Among these, vinculin acts at the core of the adhesion complex-associated mechanotransduction machinery and regulates the recruitment and release of several adhesion complex components^10^,^11^. Such regulated net addition and net loss of adhesome proteins contributes to overall adhesion complex assembly and disassembly. Thus, molecular-scale mechanosensitivity may translate into variations in adhesion complex area, and vice versa^12^,^13^, eventually correlating with cell migration^14^. However, it remains to be clarified whether and to what extent the relationship between tension and adhesion size may influence adhesion complex focality (restricted adhesion complex size) and transience (restricted adhesion complex lifetime).

Previous studies using diverse experimental systems and techniques have reported either a positive correlation between tension and adhesion complex size^15^,^16^,^17^,^18^,^19^,^20^,^21^,^22^ or a negative correlation^23^,^24^. Alternatively, the existence of a tension–adhesion complex area correlation has been suggested to be adhesion complex size^25^ or growth-phase^26^ dependent, potentially with non-linear properties^27^.

These contrasting findings raise a fundamental question: is the tension–adhesion complex area relationship in fact plastic and contextually dependent, as implied by the diversity of published findings? For example, does this relationship vary depending on the current state of a given adhesion complex? To address whether the tension–adhesion complex area relationship may be plastic and, furthermore, to what extent this relationship may explain adhesion complex characteristics (including adhesion complex focality and transience), we simultaneously mapped CMAC area and tension mediated by the vinculin-mimicking VinTS tension-probe^28^. This probe enables FRET (Förster resonance energy transfer)-based force measurement across the critical mechanosensory component vinculin^29^. Notably, this approach differs from the most obvious alternative methodology, traction-force microscopy (TFM), because force application is measured on a per molecule basis, rather than through reconstruction of forces applied onto the substrate.

As applied herein, the VinTS probe permits the correlation of molecular-scale tension dynamics and macromolecular-scale adhesion complex area dynamics from the same adhesion complex over time. To achieve this we established a novel analysis pipeline, described extensively in Fig. 1. In short, H1299 cells transfected with the VinTS probe were imaged during random migration (Step 1), with adhesion segmentation and tracking then enabling extraction of CMAC size and vinculin-mediated tension (V-tension) values over time (Step 2). Subsequently, the cross-correlation of these two signals was assessed using a moving time window (Step 3). Each cross-correlation value (per adhesion, per timepoint) was contextualized based on current adhesion complex area and V-tension values, as well as their rates of change, thereby populating conditional maps of cross-correlation probabilties (Step 4). These maps allowed simulation of adhesion behaviours (Step 5), giving rise to synthetic adhesion complex properties. These were compared with the experimentally derived empirical distributions (Step 6), providing a validation of underlying data, while also facilitating the prediction of metastable CMAC subpopulations (Step 7). A hypothetical mechanism (microtubule targeting-mediated disassembly) for the regulation of such metastable CMACs was then tested experimentally (Step 8).

We find that most adhesion complexes are subject to mechanical force-linked regulation of their size and lifetime, sufficient to explain why adhesion complexes have a limited area (focality) and lifetime (transience). In addition, a small number of adhesion complexes enter a metastable state and may require additional regulation by a microtubule-dependent mechanism for their disassembly.

## Results

### The VinTS probe measures vinculin-mediated tension

**Figure 2:**
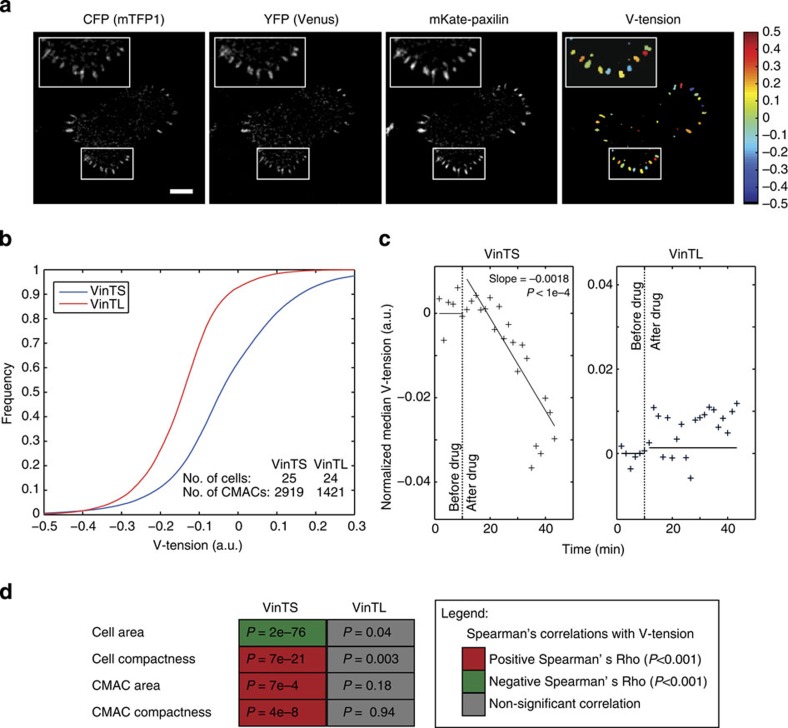
The VinTS tension sensor measures vinculin-mediated tension signals. (**a**) Representative images of a VinTS+mKate-paxilllin cell used for quantification. Channels used for imaging as indicated. Scale bar, 5 μm. (**b**) Cumulative distribution functions (CDF) of vinculin-mediated tension (V-tension) signals measured in cell-matrix adhesion complexes (CMACs) of cells transfected with tension sensor (VinTS) or the corresponding tail-less control (VinTL). The number of cells and CMACs are specified. a.u.=arbitrary units. (**c**) V-tension responses to tension inhibition using the ROCK inhibitor Y-27632. Cells were imaged every 10 s. Each data point represents the median V-tension value of all adhesions per bin of 10 time points. V-tension trends (solid lines) before and after addition of 2 μM Y-27632 (dotted vertical line) were calculated using robust linear regression. Left: VinTS cells; right: VinTL cells. V-tension values were normalized to the intercept of the fitted regression before drug addition. VinTS: 9 cells, 2194 CMACs; VinTL: 10 cells, 2206 CMACs. *P* values were calculated by robust linear regression. (**d**) V-tension measures correlate with cellular and CMAC parameters in VinTS expressing cells but not in VinTL cells. Spearman's rank correlations indicated as described in the right box. Corresponding *P* values are shown in the illustrative matrix to the left.

To concurrently assess vinculin-mediated tension (V-tension) and cell-matrix adhesion compex (CMAC) area dynamics, H1299 cells stably expressing either the tension sensor (VinTS), or the tension-insensitive tail-less control construct (VinTL)^28^, were imaged at 30 s intervals during random cell migration on fibronectin. Image analysis based on segmentation and tracking of single cells and their adhesion complex cohorts (Supplementary Fig. 1) enabled simultaneous extraction of quantitative features at molecular, macromolecular and cellular scales, including CMAC area and the fluorescence intensities used to generate the FRET signals. FRET signals were used to assess the average relative tension transmitted through VinTS molecules per CMAC, per time point (Fig. 2a). The correspondence between FRET signals and relative tension levels was confirmed via several distinct findings: (1) comparison of VinTS- and VinTL-derived signals measured within segmented adhesions revealed a clear distribution shift, indicative of VinTS probe tension-sensitivity (Fig. 2b). (2) Treatment with Y-27632 to inhibit ROCK and thereby reduce intracellular tension^30^ progressively decreased VinTS tension signals over time post drug addition, in contrast to the VinTL-probe (Fig. 2c). (3) Significant correlations were detected between VinTS tension signals and both cell and adhesion complex morphologies previously shown to be tension-responsive^14^ (Fig. 2d). Equivalent correlations to VinTL signals were absent.

Further, the expression levels of exogeneous vinculin-based constructs were below the endogeneous vinculin levels (Supplementary Fig. 2a). Importantly, the VinTS and VinTL signal distributions were similar when comparing regions outside adhesions wherein tension sensing is not expected (Supplementary Fig. 2b). In addition, the probe concentration did not substantially influence these outcomes, given that the median correlation between VinTS intensity and V-tension signals per adhesion was near zero for both VinTS and VinTL (Supplementary Fig. 2c). Moreover, even at low intensities (small or dim adhesions) we measure similar V-tension-distributions as with high-intensity objects and therefore can exclude bleed-through artefacts (Supplementary Fig. 2e).

In all, these findings confirm the tension-sensitivity of the VinTS probe, indicating that our FRET signals are indeed reflective of the tension experienced through the vinculin construct, per adhesion complex, per time point. Overall, while we observe that the VinTS sensor has a relatively low signal to noise ratio, the sampling of thousands of CMAC observations provides robust measures of vinculin-mediated tension, according to several independent criteria.

### V-tension and adhesion complex area cross-correlation

**Figure 3:**
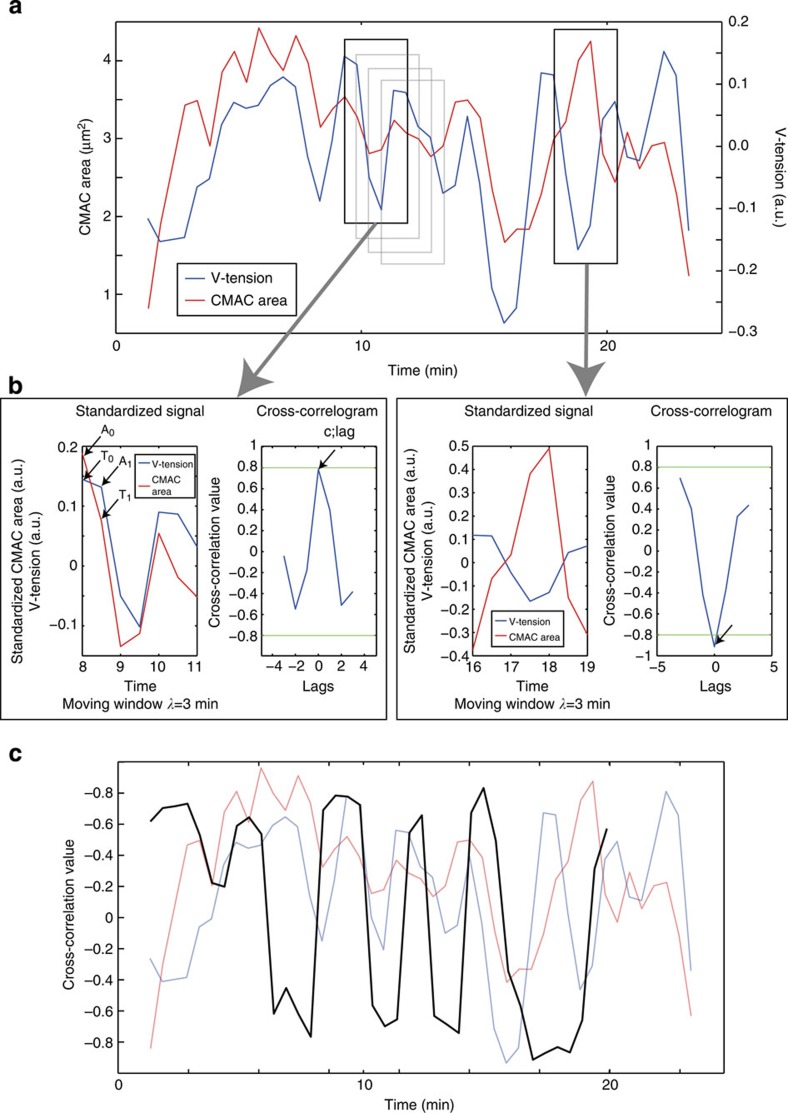
Both positive and negative cross-correlations between V-tension and CMAC area exist. (**a**) Example graph of cell-matrix adhesion complex (CMAC) area (red) and vinculin-mediated tension (V-tension; blue) over time. Black boxes represent the cross-correlation windows depicted in (**b**). The grey boxes illustrate the sequential moving windows used for analysis. (**b**) Examples of positive (left box) and negative (right box) cross-correlation of V-tension and CMAC area. For each time window, CMAC area values were standardized (by subtracting the mean CMAC area in the time window and dividing by the corresponding standard deviation of CMAC area) and cross-correlated with the V-tension. The right plot in each panel shows the cross-correlogram of the signals to the left. Apart from the cross-correlation value (**c**) and its temporal lag (lag), the initial CMAC area (A_0_), the CMAC area one time point after (A_1_), the initial V-tension value (T_0_) and the V-tension value one time point after (T_1_) were used for further calculations. Window: 6 time points (=3 min). Green lines in the cross-correlogram mark the 95% confidence bounds used as threshold to select significant cross-correlations for further analyses. (**c**) Overlay of the plot in (**a**) (in background) with the moving cross-correlation values (black).

**Figure 4:**
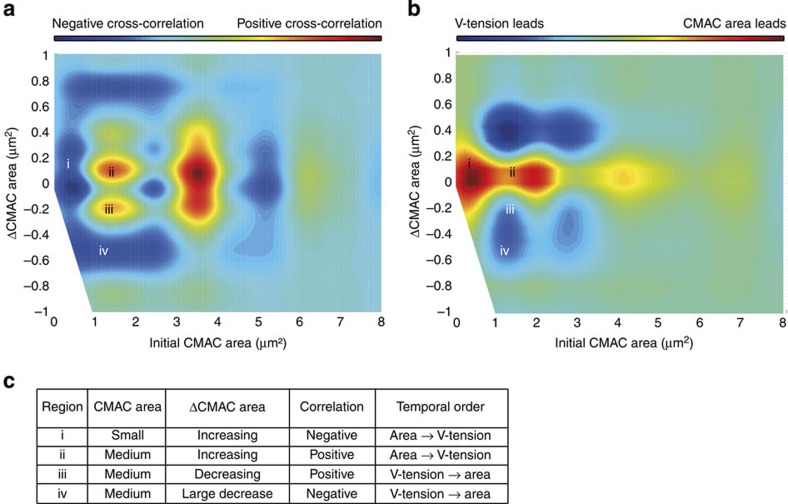
The V-tension-CMAC area relationship is plastic and has non-monotonic and multi-modal properties. (**a**) Cell-matrix adhesion complex (CMAC) area-conditioned cross-correlation net probability map showing positive (red) or negative (blue) cross-correlation between vinculin-mediated tension (V-tension) and CMAC area in the CMAC area/ΔCMAC area space. *x* axis: initial CMAC area (value A_0_ in Fig. 3b). *y* axis: change in CMAC area (ΔCMAC area; calculated as A_1_−A_0_ of Fig. 3b). Red regions indicate where positive cross-correlation is more probable; blue regions indicate where negative cross-correlation is more probable. A total of 768 CMACs from 25 VinTS expressing cells passed the confidence criterion, in contrast to only 72 CMACs in VinTL expressing cells. Regions i–iv explained in **c**. Moreover, a multimodal behaviour can only be observed in the VinTS data set, while in the VinTL data set, these trends are not present (Supplementary Fig. 5). (**b**) CMAC area-conditioned net probability map of temporal lag ordering showing where significantly correlated CMAC area (red) or V-tension (blue) signals tend to precede each other. *x* axis: initial CMAC area (value A_0_ in Fig. 3b). *y* axis: change in CMAC area (calculated as A_1_−A_0_ of Fig. 3b). Regions i–iv explained in **c**. (**c**) Examples of all four possible combinations of positive or negative cross-correlation and temporal lag orderings (regions i, ii, iii and iv marked in **a** and **b**). Region (i) ∼0.5 μm^2^ CMACs grow preceding correlated decreases in V-tension (negative cross-correlation, changes in CMAC area precede changes in V-tension). Region (ii) ∼1.5 μm^2^ CMACs grow slightly preceding V-tension increases (positive cross-correlation, changes in CMAC area precede changes in V-tension ). Region (iii) ∼1.5 μm^2^ CMACs shrink moderately following reductions in V-tension (−0.2 μm^2^) (positive cross-correlation, changes in V-tension precede changes in CMAC area). Region (iv) ∼1.5 μm^2^ CMACs shrink significantly following increases in V-tension (negative cross-correlation, changes in V-tension precede changes in CMAC area).

As noted above, reports describing the correlation between tension and adhesion complex area appear, to date, contradictory. We hypothesized that such contradictions may reflect plasticity and context-dependence in the area-tension relationship, that is, that the type of correlation may depend on the current state of the adhesion complex (for example, its size and/or growth rate). To characterize such context-dependence, we first performed a moving window cross-correlation analysis of the CMAC area and V-tension signals (Fig. 3a). Cross-correlation analyses give information about both the type of relationship between two dynamic signals (correlation or anti-correlation) and their temporal order (lag). For instance, the representative adhesion complex displayed in Fig. 3 shows both positive and negative correlations over time between CMAC area and V-tension (Fig. 3b). Crucially, we maintained links between recorded cross-correlation values and the corresponding values of CMAC area and V-tension, as well as the rates of change in these values, per adhesion, per time point (Fig. 3c). This allowed us to recognize coherent trends in cross-correlation values by populating an area-Δarea map (that is, small versus big and growing versus shrinking CMACs) with all of the correlation values (positive or negative) observed in CMACs as a function of their size (CMAC area) and growth rate (ΔCMAC area) (*area-conditioned cross-correlation map*, Fig. 4a). This revealed local tendencies towards positive or negative cross-correlation. For instance, given all adhesion complexes with a size of 1.5 μm^2^ that are slightly growing (+0.1 μm^2^), the majority of all analysed CMACs showed a positive CMAC area-V-tension cross-correlation (adhesion complex area increases with rising vinculin-mediated tension; Fig. 4a region ii). We next performed an equivalent mapping of CMAC area-V-tension cross-correlation probabilities, conditioned this time within the V-tension-ΔV-tension space (V-tension-conditioned cross-correlation map, Supplementary Fig. 4). Finally, we also mapped how the temporal order (lags) of correlated CMAC area and V-tension dynamics varied within the CMAC area/Δarea space (showing which signal tends to precede the other in different adhesions; area-conditioned Lag map, Fig. 4b).

### A plastic relationship between V-tension and adhesion size

The landscape defined in Fig. 4a indicates that the CMAC area-V-tension relationship is highly plastic, showing non-linear, non-monotonic and multimodal characteristics. This means that no simple, linear generalization can be applied, such as ‘with increasing tension, adhesions grow'. Instead, the non-linearity of these trends, combined with their local coherence, implies that this relationship is contextually dependent (on current adhesion complex area and rate of area change), but non-random. A rugged landscape also defines the temporal signal ordering of V-tension and CMAC area signals (Fig. 4b). As expected, changes in V-tension often preceded correlated changes in adhesion complex area (V-tension upstream; Fig. 4b regions iii and iv). However, surprisingly, the inverse is also true, such that changes in adhesion complex area also preceded changes in vinculin-mediated tension (V-tension downstream; Fig. 4b regions i and ii). Thus, in terms of temporal precedence, there appears to be a flexible, bidirectional and, once again, context-dependent relationship between vinculin-mediated tension and adhesion complex area.

A combinatorial view of these two maps further emphasizes the plasticity of the V-tension-CMAC area relationship. In fact, all four possible combinations of dynamics are observed, that is, positive or negative cross-correlation, with adhesion complex area either following or leading changes in vinculin-mediated tension (exemplified in regions i–iv of Fig. 4a,b, summarized in 4c).

Interestingly, both the area-conditioned cross-correlation and temporal lag maps contain specific regional trends that may represent local regulatory regimes. From the maps in Fig. 4a,b, we identified five such regional trends, for which mechanisms are considered in detail in Discussion. These include: 1) small, growing adhesions (<1 μm^2^) exhibit a negative correlation between their area and V-tension, as previously reported by Beningo *et al*.^23^ while; 2) moderately sized adhesions (>1 & <4.5 μm^2^) with moderate dynamics (change in area >−0.4 & <0.6 μm^2^), representing ∼80% of observations, tend to show positive V-tension-CMAC area correlations, as supported by a number of publications^17^,^18^,^31^. Alternatively, (3) large adhesions (>4.5 & <6 μm^2^) tend to revert to a negative correlation, which may be reflective of mechanisms limiting the adhesion complex area distribution^26^. From Fig. 4b we note that (4) large changes in adhesion complex area (>|0.2| μm^2^) tend to follow changes in vinculin-mediated tension, while (5) small increases in adhesion complex area (<0.2 μm^2^) tend to precede changes in vinculin-mediated tension, a process that may be related to ‘tugging' to probe the substrate^32^. Overall, our contextually sensitive analysis, based on new empirical data, provides a coherent framework to integrate many previously contradictory findings. Thus, we establish a uniquely comprehensive view of the plastic relationship between mechanical force sensing and adhesion state.

### Modelling of adhesion dynamics based on empirical data

**Figure 5:**
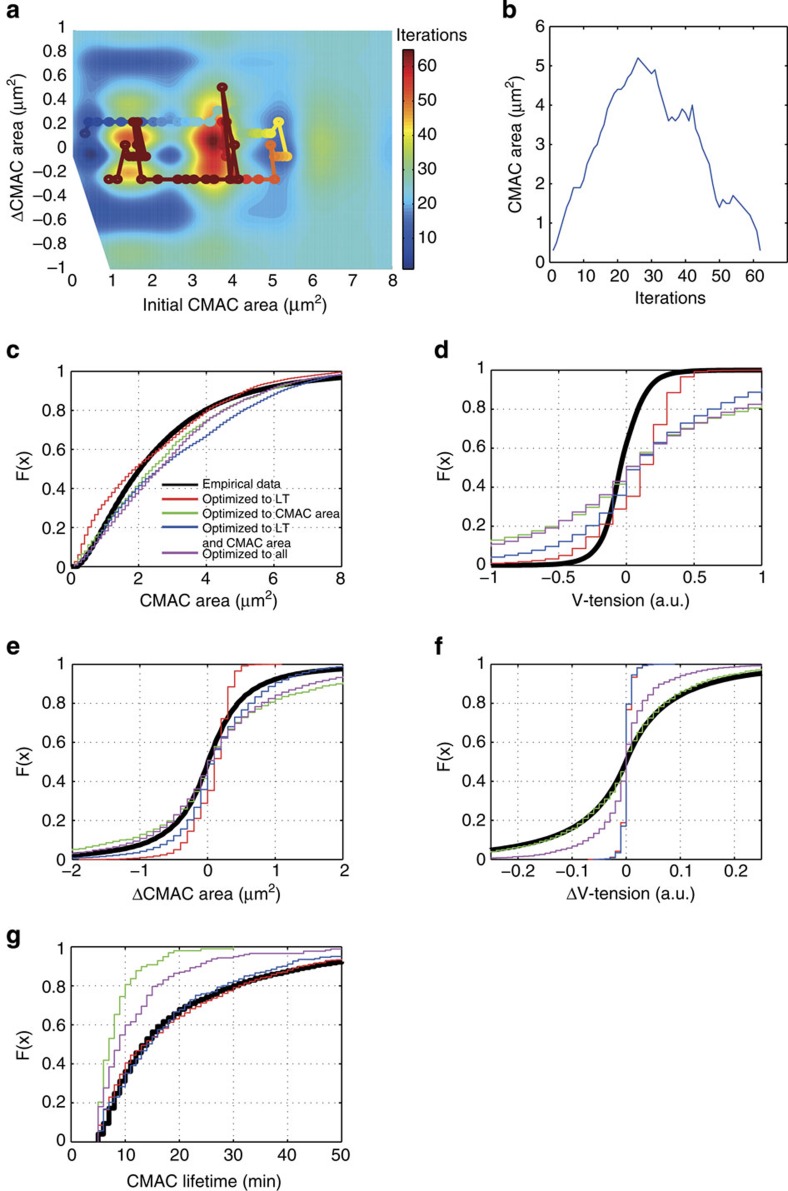
Models based on the V-tension-CMAC area relationship match empirically observed data. Modelling was performed as described in Methods and Supplementary Fig. 6 in Supplementary Notes. (**a**) Maps in Fig. 4a and Supplementary Fig. 4 were used in modelling to simulate cell-matrix adhesion complex (CMAC) behaviour using an initial CMAC area of 0.2 μm^2^ and the empirical median initial vinculin-mediated tension (V-tension) (0.1 a.u.) as a starting point. A sample synthetic CMAC trajectory within the CMAC area-conditioned cross-correlation map is displayed and colour-coded for simulation iterations. (**b**) CMAC area per iteration is shown for the same synthetic CMAC shown in **a**, wherein complete CMAC assembly and disassembly occur within 60 iterations. (**c**–**g**) Synthetic distributions for CMAC area (**c**), V-tension (**d**), ΔCMAC area (**e**), ΔV-tension (**f**), and CMAC lifetime (LT) (**g**) were plotted as cumulative distribution functions (CDFs) and compared with the empirical CMAC feature distributions (CDFs in black). Other CDF colours indicate results from selected models that best predict: the empirical lifetime distribution (red); the empirical CMAC area distribution (green); both empirical CMAC area and lifetime distributions (blue); or all five key CMAC feature empirical distributions (magenta) according to the goodness-of-fit Pearson's χ^2^ statistics.

To test the biological relevance of the two probabilistic cross-correlation maps representing the V-tension-CMAC area relationship, we performed modelling based on their combined topologies, as described in Methods and Supplementary Fig. 6 in Supplementary Notes. In figurative terms, we allowed a theoretical adhesion complex to ‘ping-pong' between these two cross-correlation landscapes, with such bouncing subtly modified by tunable stochastic variability. This modelling allowed the simulation of synthetic adhesion trajectories over iterative time points in the four-coordinate area-Δarea-V-tension-ΔV-tension space (example represented in the area-Δarea landscape, Fig. 5a). These synthetic trajectories often resemble expected empirical adhesion complex behaviours, including a growth phase, a dynamic intermediate phase and a final disassembly phase (Fig. 5b). Given this positive indication, a spectrum of 10,000 different models was developed, with each applied to generate 1,000 simulated CMAC trajectories from which population distributions describing key features of synthetic CMACs were extracted.

### Synthetic adhesion complex properties match empirical data

The accurate reconstruction of empirical distributions through modelling is likely to be possible only if cross-correlation maps are accurate representations of the true V-tension-CMAC area relationship. Excitingly, by comparing empirical and synthetically generated adhesion complex population data, we observed striking correspondences. This represents a strong validation of the complex V-tension-CMAC area relationship defined by the cross-correlation maps. To achieve this outcome, model optimization was performed by finding best-fits between the synthetic and empirical distributions for CMAC area, V-tension, Δarea, ΔV-tension and adhesion lifetime, or combinations of these features. Specifically, we now consider four models optimally fitting CMAC area, adhesion lifetime, both CMAC area and lifetime, or all five indicated distributions (Fig. 5c–g). These models closely predict the empirical distributions of CMAC area (Fig. 5c) and V-tension (Fig. 5d), features for which information is intrinsic to the model, as CMAC area and V-tension are direct input variables. However, these models were also able to predict the distributions of variables such as ΔCMAC area (Fig. 5e) and ΔV-tension (Fig. 5f), which are not direct input variables (although they are related). Remarkably, CMAC lifetime (Fig. 5g), for which no direct information is present in the model, was also predicted with high accuracy. Critically, adhesion lifetime shows no correlation with either CMAC area (Supplementary Fig. 7a,b) or with V-tension (Supplementary Fig. 7c,d), and thus it is a *de facto* independent variable. This indicates that the success of modelling and distribution optimization is highly unlikely to result from circular reasoning. Instead, the capacity of these models to reconstruct both dependent and independent feature distributions confirms the biological relevance of the probabilistic maps (Fig. 4a and Supplementary Fig. 5). Moreover, the match of synthetic and emipirical CMAC area and lifetime distributions (even when optimized to unrelated parameters) implies that the V-tension-CMAC area relationship is able to explain, to a large extent, the focal (limited adhesion complex size) and transient (limited adhesion complex lifetime) nature of adhesion populations.

In summary, we have thus far (1) defined the V-tension-CMAC area relationship as plastic, and; (2) shown that this plastic relationship is sufficient to explain and predict fundamental adhesion complex characteristics.

### V-tension-size relationship governs most adhesion behaviours

**Figure 6:**
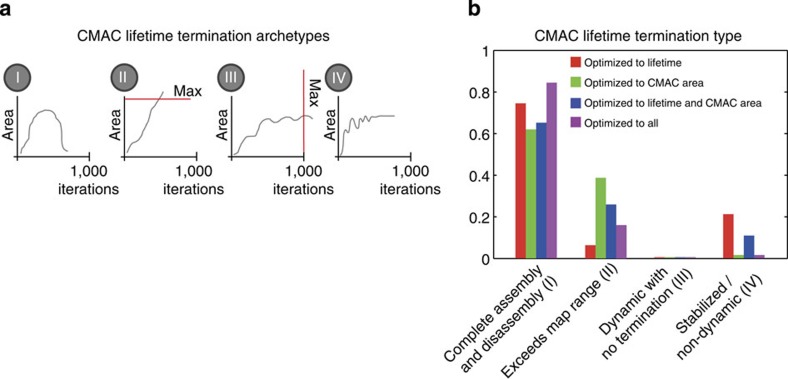
Modelling predicts a subpopulation of metastable adhesions. Schematic representation of the termination archetypes (**a**) and the relative frequency (**b**) of four possible synthetic cell-matrix adhesion complex (CMAC) termination types for the modelling: (I) the synthetic CMAC undergoes complete assembly and disassembly; (II) synthetic CMAC area or vinculin-mediated tension (V-tension) values exceed the ranges of the net-probability maps and cannot be modelled further; (III) the synthetic CMAC remains dynamic but does not terminate within 1,000 iterations of the model; (IV) synthetic CMAC area and/or V-tension values become stabilized/non-dynamic. Note: all optimized models predict a minor fraction of stabilized/non-dynamic CMACs (termination type IV).

To quantify to what extent adhesion complex behaviour could be explained by the V-tension-CMAC area relationship, we assessed what proportion of synthetic adhesions displayed complete lifetimes, including a complete disassembly phase, which is not a predetermined outcome of the models. The presence of adhesion complexes whose complete lifetimes could not be modelled would imply limits to the regulatory influence of the V-tension-CMAC area relationship, as defined herein. Alternatively, as exemplified in Fig. 5a, the generation of complete adhesion trajectories, from assembly to disassembly, implies the sufficiency of this relationship to fully explain the dynamics of a particular synthetic CMAC. Focusing on the non-determined process of disassembly, four distinct termination archetypes for adhesion trajectories are conceivable in our models (Fig. 6a): (I) simulated adhesions may assemble and disassemble completely; (II) simulated CMAC area, ΔCMAC area, V-tension, or ΔV-tension values may exceed the limits of the sampled cross-correlation maps; (III) simulated adhesions may remain dynamic yet fail to terminate within the 1,000 simulation iterations; (IV) simulated adhesions may enter a metastable state wherein CMAC area and V-tension values become non-variable. We assessed the frequencies of termination types I-IV arising from the four optimized models (Fig. 6b), finding that the vast majority (∼60 to 80%) of synthetic CMAC trajectories ends via termination type I, mimicking commonly described empirical behaviours. This indicates that the V-tension-CMAC area relationship is indeed sufficient to explain most CMAC trajectories, and therefore may govern most adhesion lifetimes. Importantly, in the four optimal models, no CMAC trajectory reached 1,000 simulation iterations (termination type III).

### Modelling predicts metastable adhesion subpopulations

Intriguingly, all four optimized models also predicted the existence of adhesion trajectories that attain a stable steady-state where CMAC area and V-tension values become equilibrated and no longer vary (Termination type IV, Fig. 6a,b). This exposes putative limits to the explanatory power of the mapped V-tension-CMAC area relationship, and predicts that subpopulations of adhesion complexes stable for CMAC area and V-tension may exist (that is, CMAC area and V-tension ‘steady-state' or ‘metastable' adhesions). Remarkably, this corresponds with recent TFM-derived empirical evidence that adhesion complexes may be divided into tension-dynamic and tension-stable subpopulations^33^.

### Metastable adhesions are enriched at specific adhesion sizes

**Figure 7:**
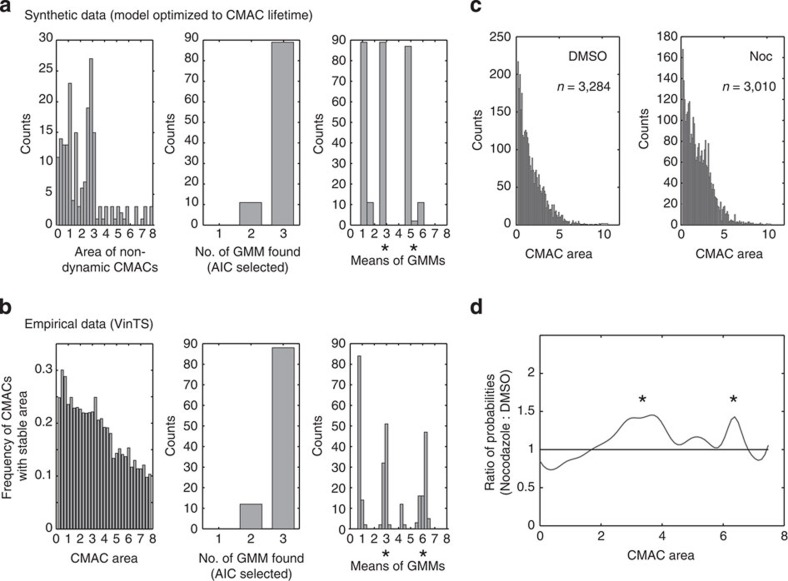
Putative metastable adhesions are selectively enriched by microtubule disruption. (**a**) The cell-matrix adhesion complex (CMAC) area distribution of synthetic non-dynamic CMACs (synthetic population from the model optimized for CMAC lifetime, shown in Fig. 6b; termination mode IV) shows a non-random distribution (left panel). Akaike information criterion (AIC)-based selection of Gaussian Mixture Models (GMMs) suggests the presence of 2 or 3 subpopulations (centre panel). Means of GMM-predicted subpopulations cluster around CMAC area values of ∼1, ∼3, and ∼5–6 μm^2^ (right panel). (**b**) The CMAC area distribution of empirical non-dynamic CMACs (CMACs that remain the same size in two consecutive timepoints) shows a non-random distribution (left panel). AIC-based selection of GMMs suggests the presence of 2 or 3 subpopulations (centre panel). Means of GMM-predicted subpopulations cluster around CMAC area values of ∼1, ∼3, and ∼6 μm^2^ (right panel). (**c**) Empirical distributions of CMAC areas from fixed VinTS-expressing cells following a 1 h DMSO (left) or nocodazole (right, 1 μM) treatment. (**d**) Ratio of the probabilities of CMAC area distributions in **c**. Microtubule disruption by nocodazole selectively enriched CMACs with areas around putative stable attractor state values of ∼3 and∼6 μm^2^, highlighted by asterisks in **a**, **b** and **d**.

To test this prediction, we studied the CMAC area distributions of synthetic metastable adhesions, which remain stable for both CMAC area and V-tension. This revealed a multimodal distribution (Fig. 7a, left). By repetitively applying Gaussian mixture modelling (GMM), a statistical tool to identify subpopulations in an unbiased manner^34^, we identified either two or three metastable adhesion complex subpopulations (frequencies shown in Fig. 7a, middle). These adhesion complex subpopulations have mean CMAC area values in the ranges of 1–2 μm^2^, 2.5–3 μm^2^ and 5–6 μm^2^ (Fig. 7a, right). Notably, similar data were found in synthetic distributions from a differently optimized model (Supplementary Fig. 8).

Given these specific predictions about the location (CMAC area value ranges) of metastable synthetic adhesion complex subpopulations, we used the same unbiased approach to test for the existence of corresponding stable subpopulations in empirical data. GMM was therefore applied to the empirical CMAC area distribution of stable adhesions (that is, showing the same area value in at least two consecutive time points) (Fig. 7b, left). Remarkably both the number of subpopulations and their area ranges closely match those of the synthetic data (Fig. 7b).

### Microtubule disruption enriches adhesions of predicted sizes

The empirical detection of steady-state adhesions, whose disassembly cannot be explained by the plastic V-tension-CMAC area relationship, implies the involvement of alternative disassembly mechanisms that may be (a) V-tension-independent (at least within our detection limits) and/or; (b) rapid and therefore below the temporal resolution (30 s) of our data. Importantly, adhesion complex disassembly is generally expected to correspond with progressive decreases in tension^19^,^20^. However, microtubule targeting-mediated catastrophic CMAC disassembly represents a specific alternative mechanism for which tension is not known to play an initiating role^35^. Furthermore, microtubule-mediated adhesion complex disassembly is extremely rapid, occurring within seconds^36^, meaning that any associated tension dynamics would be undetectable given our temporal resolution.

To test the hypothesis that microtubule-mediated catastrophic CMAC disassembly may be a selective mechanism for the termination of metastable adhesions, VinTS-expressing cells were treated with 1 μM nocodazole to disrupt microtubules. CMAC area distributions following DMSO (control) and nocodazole treatments were compared (Fig. 7c). We observed a selective enrichment of adhesion complexes with areas of ∼3 μm^2^ and ∼6 μm^2^ in nocodazole-treated cells (Fig. 7d), contributing to an overall increase in CMAC size, as previously reported^37^,^38^. Strikingly, these overrepresented CMAC area values correspond to those wherein metastable adhesions are enriched within both synthetic and empirical data sets (see asterisks in Fig. 7a right, 7b right and 7d). These data support the hypothesis that steady-state adhesion complex subpopulations may undergo swift disassembly by a microtubule-targeting-mediated mechanism, which would therefore represent a selective stimulus for steady-state exit. The combined explanatory power of the V-tension-CMAC area relationship and this alternative process of microtubule-mediated adhesion complex disassembly, targeting those adhesions in a metastable state, provides a comprehensive view of the regulatory mechanisms governing adhesion complex behaviour.

## Discussion

This study provides a comprehensive characterization of the relationship between vinculin-mediated tension (V-tension) and cell-matrix adhesion complex (CMAC) area. By extensively mapping the correlated dynamics of these variables, we generated probabilistic landscapes that contain non-linear, non-monotonic and multi-modal features, thereby defining the plastic and context-dependent nature of the V-tension-CMAC area relationship. Mechanistically, such plasticity in the V-tension-CMAC area relationship likely reflects underlying changes in the biochemical states of adhesion complexes, involving molecular-scale switches^22^. This hypothesis is supported by the tension-responsiveness of regulatory adhesion complex components (for example, vinculin, talin, FAK, paxillin, zyxin, ILK, p130Cas, vinexin^9^,^11^,^39^), whose adaptor and/or signalling activities are modulated by applied tension levels. This underlines the importance of mechanotransduction at the molecular scale in the determination of macromolecular scale adhesion properties, such as area^12^, which in turn regulate higher-order processes, such as cell migration^14^.

Remarkably, most preceding findings, though often contradictory and derived from different techniques, may now be consolidated based on the specific regional trends identified through our probabilistic mapping. This is despite the fact that our data are limited to measurements of vinculin-mediated tension, rather than total force transmission. For example, in our results, most moderately sized adhesions show positive correlations between V-tension and CMAC area, supporting numerous previous findings^17^,^18^,^31^. Yet, most small and large adhesion complexes show negative correlations, corresponding with alternate observations^23^,^24^. Thus, rather than representing contradictions, such findings may now be logically interpreted as reflecting contextual dependence upon CMAC area and ΔCMAC area values.

One of the most interesting regional trends identified herein is the negative correlation between CMAC area and V-tension found in large adhesion complexes. This represents an inversion of the positive correlation observed for moderately sized adhesion complexes. This inversion has profound implications, as it may demarcate specific mechanisms that define an upper limit to the size of adhesions. This potentially includes terminating the positive feedback-loop thought to link increases in mechanical tension with increases in adhesion complex growth^7^,^40^,^41^. Termination of this feedback-loop is likely to serve a key role in constraining the CMAC area distribution, as well as triggering adhesion complex disassembly and, thus, also limiting adhesion complex lifetime. Consequently, this particular regulatory regime may be critical for the definition of adhesion complex focality (limited CMAC area) and transience (limited CMAC lifetime). Notably, although our findings specify negative correlation rather than V-tension-independence, they bare similarity to previous indications that, after reaching large sizes, adhesions no longer grow in response to increased tension^26^.

In addition to mapping cross-correlation probabilities, our analysis uniquely resolves the temporal ordering of spontaneous V-tension- and CMAC area-dynamics to establish an unprecedentedly complete picture of tension-linked adhesion complex behaviours. As implied by previous perturbation studies^42^, changes in tension often precede changes in CMAC area, most likely in a causal manner. Indeed, we observed that large changes in CMAC area predominantly occurred following changes in V-tension. This implies that V-tension fluctuations at the molecular level can profoundly influence adhesion complex structure at the macromolecular scale. Surprisingly, however, we also observed that small increases in adhesion area typically preceded changes in vinculin-mediated tension. Explicitly, in small, growing adhesion complexes, wherein V-tension and CMAC area tend to be negatively correlated, slight increases in area may act to promote the redistribution of forces across additional load-bearing molecules as they are incorporated into the adhesion complex. This would dictate decreased tension levels per molecule, even though total tension applied to the adhesion complex may increase^22^. This hypothesis is especially attractive for immature adhesion complexes with limited F-actin connectivity, whose initial growth is thought to be biochemically rather than mechanically driven^43^,^44^,^45^. In contrast, within moderately sized adhesion complexes, small increases in CMAC area before correlated V-tension increases may reflect pre-emptive tuning of load-bearing structures in preparation for subsequent increases in mechanical stress. Together, these unique, temporally resolved findings emphasize that, although tension (molecular-scale dynamics) usually drives major CMAC area changes (macromolecular-scale adhesion complex dynamics), area changes may also, unexpectedly, precede tension responses.

Moving beyond independent interpretations of local topographic features within the relationship maps, modelling provided the means to (1) validate and (2) interpret the collective implications of these maps. First, by synthetically recapitulating the empirical distributions of CMAC feature values (including features independent from modelling inputs, that is, CMAC lifetime), modelling confirmed that the correlation probability maps meaningfully represent the V-tension-CMAC area relationship. Second, assessing the proportion of synthetic adhesion complexes that terminate via disassembly (termination type 1) indicated that the V-tension-CMAC area relationship (as defined) is sufficient to explain the complete lifetime dynamics of most (60–80%) individual adhesions. This relationship is thus a major determinant of parameter distributions (including CMAC area and lifetime) at the adhesion population level. We therefore conclude that this relationship plays a vital role in shaping fundamental adhesion complex properties, such as adhesion complex focality and transience.

While the modelling approach applied herein did recapitulate empirical adhesion population properties with high accuracy, it could not fully account for the properties and dynamics of all individual adhesions. Specifically, the disassembly of a small proportion of synthetic adhesion complexes remained unexplained, because in each of our optimized models, some adhesions stabilized to a metastable state wherein V-tension and area values remained unchanged over time. Given that all empirical adhesions do disassemble, this result highlights a specific limit to the explanatory power of the V-tension-CMAC area relationship, as currently defined. Nonetheless, these modelling-based results precipitated two important biological predictions: (a) that equilibrated, steady-state adhesion subpopulations may exist and; (b) that these adhesion complexes may require a specialized disassembly mechanism that our data collection failed to adequately capture, potentially because it is rapid and/or V-tension-independent. Such an alternative disassembly mechanism is necessary to explain how these metastable adhesion complexes terminate their lifetime, given that measured changes in vinculin-mediated tension and CMAC area are unable to do so.

Steady-state adhesion complexes were identified in both synthetic and empirical adhesions using an unbiased approach to subpopulations detection. This analysis clearly and reproducibly indicated the localized enrichment of metastable adhesions at specific sizes, with a near perfect correspondence between the area values predicted in empirical and synthetic CMAC data. This suggests the existence of underlying ‘attractor' states in our probabilistic landscapes. In the context of complex systems, attractor states represent organizational states of a system where an equilibrium is more likely to arise, leading to the local enrichment of steady-state observations^46^. Notably, our finding that most individual adhesions display dynamic V-tension levels, while a small proportion display stable V-tension values, is in accordance with recent TFM-derived data showing distinct subpopulations of stable and dynamic adhesions, according to force measurement^33^.

Given evidence supporting the existence of steady-state adhesion subpopulations (confirming the first prediction, above), we next addressed the second prediction by attempting to identify a specific disassembly mechanism that is either rapid and/or V-tension-independent, and therefore not detected in our current data. Notably, microtubule targeting-mediated catastrophic CMAC disassembly is a suitable candidate mechanism, given that it is selective^35^,^37^, rapid (occurring in few seconds^47^, well under our 30 s resolution), and initially tension-independent (with tension responses likely being a downstream response rather than the initiating mechanism^37^,^48^). Accordingly, by using nocodazole to disrupt microtubules, we observed significant enrichment of adhesion complexes in the same CMAC area ranges where metastable adhesions were over-represented in both synthetic and empirical adhesion population data. The specificity of this enrichment strongly supports a selective role for microtubule targeting-mediated adhesion complex disassembly in the termination of stable, attractor state-locked adhesions. This selective mechanism would differ from, for example, indirect effects on global tension^49^, which would be expected to have more general (not selectively enriched) effects on CMAC area distributions. Thus, our data lead to the prediction that the well studied mechanism of microtubule targeting-mediated catastrophic CMAC disassembly is in fact selective for a specific subpopulation of steady-state adhesions.

Despite significant technical and analytical advances, our analysis is still constrained somewhat by the limited dynamic range (approximately 1–6 pN) and low signal to noise ratio of the VinTS probe. The limited dynamic range dictates that CMACs experiencing tension fluctuations outside these values will not give a significant cross-correlation value and are therefore not captured in the relationship maps. Similarly, low signal to noise may ultimately obscure instances of cross-correlation, although there is no indication that this would occur in a biased manner, and hence this may not substantially alter our current interpretations. Nonetheless, in light of these key limitations, it is worth noting that the V-tension-CMAC area relationship as currently defined could (a) contain a mixture of differently behaving adhesion subpopulations; (b) hide less abundant but different adhesion behaviours, and/or; (c) be more nuanced given higher spatiotemporal resolution, higher signal to noise ratios and a higher dynamic range.

Overall, in this study, we describe a novel framework for conceptualizing and exploring adhesion complex regulation. Extensive mapping of the correlative links between vinculin-mediated tension and CMAC area revealed a plastic and context-dependent relationship. Modelling suggested that a majority of adhesions are principally regulated by mechanisms encapsulated by the V-tension-CMAC area relationship, dictating essential properties of adhesion complex populations (focality and transience). Modelling also unexpectedly predicted a subpopulation of steady-state, attractor-associated adhesions for which evidence was also detected in empirical adhesion populations. Finally, we indicated that the disassembly of metastable adhesions may be selectively induced by microtubule targeting. We thus present a comprehensive interpretation of the balance between mechanotransduction- and microtubule-based mechanisms of adhesion complex regulation.

## Methods

### Cell culture and reagents

H1299 human non-small lung cancer cells (kind gift from B. Geiger, The Weizmann Institute of Science, Israel) were cultured in RPMI-1,640 (Gibco) medium supplemented with 10% FBS (Gibco) and 5 mg/ml L-Glutamine (Gibco).

Expression plasmids used include the previously described VinTS tension sensor and VinTL tail-less control (constant FRET)^28^; (kind gifts from M.A. Schwartz, University of Virginia, USA). The pGL4.21 vector and the mKate2-paxillin plasmid (kindly gifted by K. Lukyanov, Institute of Bio-organic Chemistry, Moscow, Russia) were also employed. Fibronectin was purified from human blood serum according to previously described methods^50^.

Stable cell lines expressing the VinTS tension sensor or VinTL control constructs were generated as follows: H1299 cells were co-transfected with the VinTS or VinTL plasmids, the mKate2-Paxillin construct and the pGL4.21 vector containing a Puro^R^ cassette, using Lipofectamine Plus (Invitrogen) according to the manufacturer's instructions; 48 h after transfection, cells were subjected to selection with puromycin (2 μg/ml, Sigma) and subsequently FACS sorted (FACS ARIA, BD). Cells were cultured in the presence of the selection antibiotic.

To disrupt microtubule function, cells were treated with 1 μM nocodazole (Sigma) or DMSO (1:16,667) for 1 h, and subsequently fixed (4% PFA in PBS). To inhibit ROCK signalling, cells were treated with Y-27632 (2 μM; Sigma).

### Immunoblot

H1299 cells were detached with EDTA 2 mM in PBS and lysed in a buffer with 0.5% NP-40, 50 mM Tris-HCl pH 7.4, 150 mM NaCl and 1 mM EDTA. Cell extracts were subjected to SDS–PAGE, followed by a semi-dry transfer to an Immobilon (Millipore) membrane and immunoblotting with primary antibodies anti-vinculin (clone hVin-1; Sigma; #V9131 1:2,000), and anti GAPDH (clone 6C5; Millipore; #MAB374; 1:5,000; used as loading control). Subsequently, incubation with horseradish peroxidase-conjugated secondary antibodies (Jackson; #715-035-151; 1:3,000) enabled protein detection with chemiluminiscent substrate (Pierce; ECL plus; #32132-P).

### Live-cell ratiometric imaging

PBS-washed optical glass-bottomed 96-well plates (Matrical) were coated with fibronectin (10 μg ml^−1^) in PBS at 37 °C for 1 h, followed by blocking with 0.5% heat-denatured BSA (Sigma) in PBS for 20 min, and RPMI-1640 washing (3 × ). Cells were trypsinised, PBS-washed and replated in RPMI-1640+0.1% FBS into the fibronectin-coated plates (1,500 cells/well). Cells were then incubated for 4 h. Live-cell imaging was performed using a Nikon A1R confocal under cell-culture conditions. Images were acquired every 30 s for 4 h, using a × 60 Plan Apo oil objective (1.4 NA), × 1.25 zoom and 512 × 512 resolution. Use of the spectral detector allowed the following optical configuration for simultaneous channel acquisition: ‘mTFP1'-channel (457 nm laser excitation, emission bandwidth collected 470–510 nm); ‘FRET'-channel (457 nm laser excitation, emission bandwidth collected 530–550 nm); ‘mKate2'-channel (561 nm laser excitation, emission bandwidth collected 600–680 nm). To detect cell responses to ROCK inhibition, cells were imaged every 10 s for 45 min, with 2 μM Y-27632 (Sigma) delivered after 55 imaging time points.

### Image processing and segmentation

All images were filtered using the smoothing, edge preserving bilateral filter^51^ (Matlab function written by Douglas R. Lanman, Brown University; dlanman@brown.edu). The following parameters were visually adjusted and applied to all channels: Gaussian bilateral window half-size (defined as 7 pixels); Bilateral filter s.d. values (1 pixel for both the spatial and the intensity domains).

Patch Morphology Analysis Dynamic software (Digital Cell Imaging Laboratories, Belgium) was used to segment and track individual cells and their cell-matrix adhesion complex (CMAC) cohorts, allowing the extraction of a spectrum of quantitative features, including CMAC size and channel intensities within each adhesion complex. Tracking-parameters applied: interpolation 1 time point; maximum step-size 3 μm; minimum track lifetime 4 time points; minimal object size 3 px (0.3 μm^2^). Segmentation masks were used to obtain pixel intensities from both CMACs and cells.

### FRET ratio calculation and vinculin-tension signal display

To calculate FRET signals from the VinTS and VinTL probes, ratios were generated between Venus and mTFP1 mean intensities, per CMAC. Ratios were linearized by logarithmic transformation and inverted to more intuitively represent relative tension signal levels (since high vinculin-mediated tension (V-tension) corresponds to low FRET). Thus, the measure that we describe here as ‘vinculin-mediated tension' or V-tension reflects the average relative V-tension transmitted through the exogenous vinculin-based tension sensor molecules in a segmented object.

To assess the influence of potential channel bleed-through or cross-activation, we performed spectral imaging of fixed VinTL and VinTS expressing cells, followed by spectral unmixing based on single fluorophore spectra obtained under equivalent experimental and optical conditions. No difference in ratio measurements was observed between standard acquisition or spectrally unmixed acquisition. Thus, the contributions of potential bleed-through or cross-activation were considered negligible in our system.

To estimate the sensor concentration, an average signal of the mTFP1 and Venus channel was generated, which we refer to as ‘CY'-signal.

### Cross-correlation analysis

A smoothing spline algorithm implemented as a MATLAB function (DeBoor's algorithm; Fred Frigo; http://www.eng.mu.edu/frigof/spline.html) was applied to CMAC Area, CMAC Mean Intensity CY and V-tension time series. The smoothing factor for all signals (value exp (−10^−6^)) was visually selected. Delta values of these parameters were calculated after smoothing. For further processing, only CMACs with lifetimes >5 time points and a minimal absolute range of CMAC area variation greater than 0.5 μm^2^ were considered.

Cross-correlation was used to leverage time-resolved information contained in the synchronous dynamics of CMAC area and V-tension over individual CMAC lifetimes. Short time windows (Δ=3 min) were selected to link changing cross-correlation values over time to the changing state of each CMAC (Fig. 3a). For each moving of that window the initial CMAC area (at time 0, A_0_), the change in area (from time 0 to 1, Δarea, A_1_–A_0_), the initial V-tension (T_0_) and the change in V-tension (ΔV-tension, T_1_–T_0_) were determined. CMAC area and V-tension distributions were then standardized by subtracting the window's mean and dividing it by the maximal s.d. of VinTL and VinTS parameter distribution. Significant cross-correlation values (*r*>|0.8|) and their temporal lags (time offset between correlated signals) were also extracted (Fig. 3b: left box, positive V-tension-CMAC area cross-correlation; right box, negative V-tension-CMAC area cross-correlation). Notably, 768 CMACs showed a significant cross-correlation signal in VinTS expressing cells, while signal was found only in 72 CMACs in VinTL expressing cells.

### Cross-correlation probability map generation

Cross-correlation values were binarized. Probability maps for both positive and negative CMAC area-V-tension cross-correlation were generated using Kernel smoothing function-estimates of (I) area versus Δarea versus positive cross-correlation; (II) area versus Δarea versus negative cross-correlation; (III) V-tension versus ΔV-tension versus positive cross-correlation and; (IV) V-tension versus ΔV-tension versus negative cross-correlation. The sum of the probabilities (maps I and II, or III and IV, respectively) were set to 1 and subtracted from each other resulting in two V-tension -CMAC area cross-correlation net probability maps for either Area versus Δarea or V-tension versus ΔV-tension (Fig. 4a and Supplementary Fig. 4). Our sensitivity to short and/or weak cross-correlation patterns is limited by the 30 s data-sampling rate, the defined time window, and the stringent statistical threshold (*α*=5%) for significant cross-correlation.

### Cell-matrix adhesion complex modelling

Our modelling was performed as follows below and is visualized in Supplementary Fig. 6 in Supplementary Notes.

The V-tension- and CMAC area-conditioned cross-correlation net probability maps collectively represent a 4-dimensional coordinate system. To initiate synthetic CMAC lifetimes, we estimated the initial values from empirical data, mimicking a small CMAC (initial area=0.2 μm^2^) that is growing (ΔArea=0.1 μm^2^) with an initial V-tension of 0.1 a.u. These values define coordinates in the CMAC area-conditioned net probability map, enabling the estimation of the net probability of a positive or negative cross-correlation between CMAC area and V-tension. This cross-correlation probability is translated into a V-tension value by multiplying it by a conversion factor (*Φ1*), which is generated as a normal random number with mean *μ1* and a standard deviation *σ1=μ1/ν* where *ν* is a noise factor and thus the stochastic component of the model. The product of this multiplication renders a modelled V-tension value for the next iteration. Given the known initial V-tension and the modelled V-tension value, a ΔV-tension value is calculated. Subsequently, the cross-correlation probability of the new coordinates in the V-tension-conditioned net probability map is similarly converted using a second conversion factor (*Φ2*), with *μ2* and *σ2=μ2/ν*. This process was iterated for up to 1,000 times, resulting in synthetic CMAC trajectories. Simulations could terminate in four ways: (1) after a complete cycle of assembly and disassembly; (2) when the modelled values for CMAC area or V-tension exceed the limits of net probability map ranges; (3) if the maximal number of iterations was reached or; (4) when CMAC area and/or V-tension values become non-dynamic. Termination type 4 was enacted when the CMAC area value did not change more than 0.1 μm^2^ in four consecutive iterations (this always implied a permanent stabilization). Given preliminary findings, we introduced time variables (τ) to the modelling applied herein, defining the number of iterations before termination of simulations that remain non-dynamic: *τ1* was applied for CMAC areas <2 μm^2^; *τ2* for CMAC areas between ⩾2 μm^2^ and <3.5 μm^2^; and *τ3* for CMAC areas ⩾3.5 μm^2^. Thus, the following parameters were used to create different models.

*μ1*=[0.001 0.025 0.005 0.0075];

*μ2*=[0.025 0.05 0.1 0.5 0.75];

*ν*=[0.0625 0.125 0.25 0.5];

*τ1*=[1 2 3 5 10];

*τ2*=[1 2 3 5 10];

*τ3*=[1 2 3 5 10];

By iterating through this 6-dimensional array, we simulated a total of 10,000 distinct models, each generating 1,000 synthetic CMAC trajectories with theoretical maximal lifetimes of 1,000 iterations.

For each of the 10,000 models, the following CMAC feature distributions were extracted: CMAC area, V-tension, ΔCMAC area, ΔV-tension, CMAC lifetime (number of iterations). These synthetic distributions were compared with the empirical distributions of these features using the Pearson's *χ*^2^ goodness-of-fit test, and those achieving distributions with the minimal *χ*^2^-value were defined as optimally fitting.

### Statistics

Standard statistical techniques were implemented in MATLAB. Applied tests are mentioned in each figure. Linear trends were assessed by robust regression using the bisquare weighting function. Correlations were tested by Spearman's rank correlation. Distributions were compared using the Pearson's χ^2^ goodness-of-fit test. Differences in distribution medians were tested by Kruskal–Wallis test. Multiple comparison corrections were applied according to Tukey's honest significant difference criterion. Gaussian mixture parameters were estimated using an Expectation Maximization algorithm. Optimal number of Gaussian models were chosen based on the minimal Akaike information criterion (AIC).

## Additional information

**How to cite this article:** Hernández-Varas, P. *et al*. A plastic relationship between vinculin-mediated tension and adhesion complex area defines adhesion size and lifetime. *Nat. Commun.* 6:7524 doi: 10.1038/ncomms8524 (2015).

## Supplementary Material

Supplementary Information Supplementary Figures 1-8

## Figures and Tables

**Figure 1:**
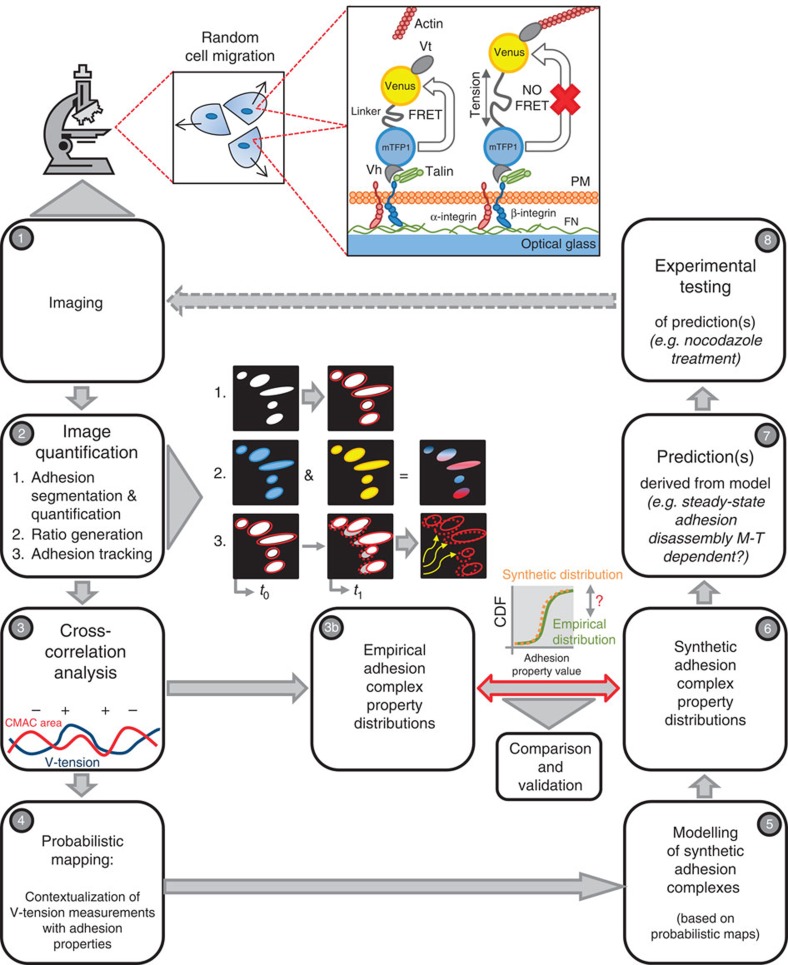
Schematic view of the experimental and analytical approach. Our analytical approach is based on quantitative microscopy and includes a systems biology-based iterative cycle involving experiments, quantitative analysis and modelling; an approach referred to as systems microscopy^52^. Steps used in this study are as follows: (1) randomly migrating H1299 cells expressing the vinculin-based tension FRET-sensor VinTS (or control VinTL)^28^ plated on fibronectin (FN) were imaged. As indicated in inset, the VinTS sensor FRETs under low tension conditions. However, under high tension, FRET efficiency drops. PM, plasma membrane. (2) Images were filtered and cell-matrix adhesion complexes (CMACs) were segmented (2.1) and their properties (including area, intensity and dynamics) extracted. FRET ratios were calculated for each CMAC and converted into vinculin-mediated tension (V-tension) (2.2). The CMACs were tracked over time (2.3). (3) For each individually tracked adhesion complex, cross-correlation analysis was performed over time (using moving windows) between V-tension and CMAC area. (3b) Data quantification (CMAC area; V-tension; their rates of change; lifetime) produces empirical adhesion complex property distributions, exemplified here by a cumulative distribution function (CDF) plot. (4) Cross-correlation values were aggregated according to their corresponding CMAC area and ΔCMAC area values. For each coordinate in the CMAC area/ΔCMAC area space, the net probability of a positive or negative cross-correlation was estimated. Similarly, a probabilistic map conditioned for V-tension/ΔV-tension was generated. These maps are quantitative representations of the V-tension-CMAC area relationship. (5) To validate this representation of the V-tension-CMAC area relationship, we tested whether empirical CMAC population property distributions could be reconstructed by stochastic modelling based on the probabilistic maps. Modelling was based on iterative determination of coordinates in the CMAC area/CMAC Δarea and V-tension /ΔV-tension spaces, incorporating a stochastic component. Each model run generates an individual synthetic CMAC trajectory. (6) Populations of synthetic CMAC trajectories generated by modelling produce synthetic adhesion complex property distributions (including area; V-tension; their rates of change; adhesion lifetime). These synthetic distributions can be compared with the empirical distributions (see 3b). As empirical and synthetic distributions matched to a large extent, we infer that the probabilistic maps on which models are based provide valid and meaningful representations of the relationship between CMAC area and V-tension. (7) Our modelling also gave rise to the novel hypothesis that a small proportion of CMACs reach a steady-state for V-tension and CMAC area. This implies that these metastable adhesion complexes may require alternative disassembly mechanisms, such as microtubule-mediated catastrophic disassembly. (8) This prediction was experimentally tested. By treating cells with nocodazole to disrupt microtubules, we observed an enrichment of CMACs within size ranges preferentially occupied by steady-state adhesions in both synthetic and empirical populations. This enrichment suggests microtubule-mediated adhesion complex disassembly as a selective mechanism for the termination of CMACs locked in a stable steady state.

## References

[b1] Webb D. J. & Horwitz A. F. New dimensions in cell migration. Nat. Cell Biol. 5, 690–692 (2003). 12894172 10.1038/ncb0803-690

[b2] Paszek M. J. & Weaver V. M. The tension mounts: mechanics meets morphogenesis and malignancy. J. Mammary Gland Biol. Neoplasia 9, 325–342 (2004). 15838603 10.1007/s10911-004-1404-x

[b3] Engler A. J., Sen S., Sweeney H. L. & Discher D. E. Matrix elasticity directs stem cell lineage specification. Cell 126, 677–689 (2006). 16923388 10.1016/j.cell.2006.06.044

[b4] van Dijk M., Goransson S. A. & Stromblad S. Cell to extracellular matrix interactions and their reciprocal nature in cancer. Exp. Cell Res. 319, 1663–1670 (2013). 23419246 10.1016/j.yexcr.2013.02.006

[b5] Hynes R. O. Integrins: bidirectional, allosteric signaling machines. Cell 110, 673–687 (2002). 12297042 10.1016/s0092-8674(02)00971-6

[b6] Lock J. G., Wehrle-Haller B. & Stromblad S. Cell-matrix adhesion complexes: master control machinery of cell migration. Semin. Cancer Biol. 18, 65–76 (2008). 18023204 10.1016/j.semcancer.2007.10.001

[b7] Kuo J. C. Mechanotransduction at focal adhesions: integrating cytoskeletal mechanics in migrating cells. J. Cell Mol. Med. 17, 704–712 (2013). 23551528 10.1111/jcmm.12054 PMC3823174

[b8] Winograd-Katz S. E., Fassler R., Geiger B. & Legate K. R. The integrin adhesome: from genes and proteins to human disease. Nat. Rev. Mol. Cell Biol. 15, 273–288 (2014). 24651544 10.1038/nrm3769

[b9] Moore S. W., Roca-Cusachs P. & Sheetz M. P. Stretchy proteins on stretchy substrates: the important elements of integrin-mediated rigidity sensing. Dev. Cell 19, 194–206 (2010). 20708583 10.1016/j.devcel.2010.07.018 PMC5319208

[b10] Carisey A. & Ballestrem C. Vinculin, an adapter protein in control of cell adhesion signalling. Eur. J. Cell Biol. 90, 157–163 (2011). 20655620 10.1016/j.ejcb.2010.06.007 PMC3526775

[b11] Carisey A. . Vinculin regulates the recruitment and release of core focal adhesion proteins in a force-dependent manner. Curr. Biol. 23, 271–281 (2013). 23375895 10.1016/j.cub.2013.01.009 PMC3580286

[b12] Parsons J. T., Horwitz A. R. & Schwartz M. A. Cell adhesion: integrating cytoskeletal dynamics and cellular tension. Nat. Rev. Mol. Cell Biol. 11, 633–643 (2010). 20729930 10.1038/nrm2957 PMC2992881

[b13] Wehrle-Haller B. Structure and function of focal adhesions. Curr. Opin. Cell Biol. 24, 116–124 (2012). 22138388 10.1016/j.ceb.2011.11.001

[b14] Lock J. G. . Plasticity in the macromolecular-scale causal networks of cell migration. PLoS ONE 9, e90593 (2014). 24587399 10.1371/journal.pone.0090593 PMC3938764

[b15] Saez A., Buguin A., Silberzan P. & Ladoux B. Is the mechanical activity of epithelial cells controlled by deformations or forces? Biophys. J. 89, L52–L54 (2005). 16214867 10.1529/biophysj.105.071217 PMC1367004

[b16] Pelham R. J. Jr. & Wang Y. Cell locomotion and focal adhesions are regulated by substrate flexibility. Proc. Natl Acad. Sci. USA 94, 13661–13665 (1997). 9391082 10.1073/pnas.94.25.13661 PMC28362

[b17] Riveline D. . Focal contacts as mechanosensors: externally applied local mechanical force induces growth of focal contacts by an mDia1-dependent and ROCK-independent mechanism. J. Cell Biol. 153, 1175–1186 (2001). 11402062 10.1083/jcb.153.6.1175 PMC2192034

[b18] Balaban N. Q. . Force and focal adhesion assembly: a close relationship studied using elastic micropatterned substrates. Nat. Cell Biol. 3, 466–472 (2001). 11331874 10.1038/35074532

[b19] Chrzanowska-Wodnicka M. & Burridge K. Rho-stimulated contractility drives the formation of stress fibers and focal adhesions. J. Cell Biol. 133, 1403–1415 (1996). 8682874 10.1083/jcb.133.6.1403 PMC2120895

[b20] Rottner K., Hall A. & Small J. V. Interplay between Rac and Rho in the control of substrate contact dynamics. Curr. Biol. 9, 640–648 (1999). 10375527 10.1016/s0960-9822(99)80286-3

[b21] Nicolas A., Geiger B. & Safran S. A. Cell mechanosensitivity controls the anisotropy of focal adhesions. Proc. Natl Acad. Sci. USA 101, 12520–12525 (2004). 15314229 10.1073/pnas.0403539101 PMC515090

[b22] Shemesh T., Geiger B., Bershadsky A. D. & Kozlov M. M. Focal adhesions as mechanosensors: a physical mechanism. Proc. Natl Acad. Sci. USA 102, 12383–12388 (2005). 16113084 10.1073/pnas.0500254102 PMC1194898

[b23] Beningo K. A., Dembo M., Kaverina I., Small J. V. & Wang Y. L. Nascent focal adhesions are responsible for the generation of strong propulsive forces in migrating fibroblasts. J. Cell Biol. 153, 881–888 (2001). 11352946 10.1083/jcb.153.4.881 PMC2192381

[b24] Gad A. K. . Rho GTPases link cellular contractile force to the density and distribution of nanoscale adhesions. FASEB J 26, 2374–2382 (2012). 22371528 10.1096/fj.11-195800

[b25] Tan J. L. . Cells lying on a bed of microneedles: an approach to isolate mechanical force. Proc. Natl Acad. Sci. USA 100, 1484–1489 (2003). 12552122 10.1073/pnas.0235407100 PMC149857

[b26] Stricker J., Aratyn-Schaus Y., Oakes P. W. & Gardel M. L. Spatiotemporal constraints on the force-dependent growth of focal adhesions. Biophys. J. 100, 2883–2893 (2011). 21689521 10.1016/j.bpj.2011.05.023 PMC3123981

[b27] Goffin J. M. . Focal adhesion size controls tension-dependent recruitment of alpha-smooth muscle actin to stress fibers. J. Cell Biol. 172, 259–268 (2006). 16401722 10.1083/jcb.200506179 PMC2063555

[b28] Grashoff C. . Measuring mechanical tension across vinculin reveals regulation of focal adhesion dynamics. Nature 466, 263–266 (2010). 20613844 10.1038/nature09198 PMC2901888

[b29] Chang C. W. & Kumar S. Vinculin tension distributions of individual stress fibers within cell-matrix adhesions. J. Cell Sci. 126, 3021–3030 (2013). 23687380 10.1242/jcs.119032 PMC3711198

[b30] Amin E. . Rho-kinase: regulation, (dys)function, and inhibition. Biol. Chem. 394, 1399–1410 (2013). 23950574 10.1515/hsz-2013-0181 PMC5538733

[b31] Delanoe-Ayari H., Al Kurdi R., Vallade M., Gulino-Debrac D. & Riveline D. Membrane and acto-myosin tension promote clustering of adhesion proteins. Proc. Natl Acad. Sci. USA 101, 2229–2234 (2004). 14982992 10.1073/pnas.0304297101 PMC356933

[b32] Plotnikov S. V. & Waterman C. M. Guiding cell migration by tugging. Curr. Opin. Cell Biol. 25, 619–626 (2013). 23830911 10.1016/j.ceb.2013.06.003 PMC3827722

[b33] Plotnikov S. V., Pasapera A. M., Sabass B. & Waterman C. M. Force fluctuations within focal adhesions mediate ECM-rigidity sensing to guide directed cell migration. Cell 151, 1513–1527 (2012). 23260139 10.1016/j.cell.2012.11.034 PMC3821979

[b34] Everitt B. S. An introduction to finite mixture distributions. Stat. Methods Med. Res. 5, 107–127 (1996). 8817794 10.1177/096228029600500202

[b35] Kaverina I., Krylyshkina O. & Small J. V. Microtubule targeting of substrate contacts promotes their relaxation and dissociation. J. Cell Biol. 146, 1033–1044 (1999). 10477757 10.1083/jcb.146.5.1033 PMC2169483

[b36] Efimov A. & Kaverina I. Significance of microtubule catastrophes at focal adhesion sites. Cell Adh. Migr. 3, 285–287 (2009). 19483470 10.4161/cam.3.3.8858 PMC2712812

[b37] Ezratty E. J., Partridge M. A. & Gundersen G. G. Microtubule-induced focal adhesion disassembly is mediated by dynamin and focal adhesion kinase. Nat. Cell Biol. 7, 581–590 (2005). 15895076 10.1038/ncb1262

[b38] Colo G. P. . Focal adhesion disassembly is regulated by a RIAM to MEK-1 pathway. J. Cell Sci. 125, 5338–5352 (2012). 22946047 10.1242/jcs.105270

[b39] Wolfenson H., Bershadsky A., Henis Y. I. & Geiger B. Actomyosin-generated tension controls the molecular kinetics of focal adhesions. J. Cell Sci. 124, 1425–1432 (2011). 21486952 10.1242/jcs.077388 PMC3078811

[b40] Yeung T. . Effects of substrate stiffness on cell morphology, cytoskeletal structure, and adhesion. Cell Motil. Cytoskeleton 60, 24–34 (2005). 15573414 10.1002/cm.20041

[b41] Maruthamuthu V., Sabass B., Schwarz U. S. & Gardel M. L. Cell-ECM traction force modulates endogenous tension at cell-cell contacts. Proc. Natl Acad. Sci. USA 108, 4708–4713 (2011). 21383129 10.1073/pnas.1011123108 PMC3064395

[b42] Bershadsky A., Kozlov M. & Geiger B. Adhesion-mediated mechanosensitivity: a time to experiment, and a time to theorize. Curr. Opin. Cell Biol. 18, 472–481 (2006). 16930976 10.1016/j.ceb.2006.08.012

[b43] Tadokoro S. . Talin binding to integrin beta tails: a final common step in integrin activation. Science 302, 103–106 (2003). 14526080 10.1126/science.1086652

[b44] Humphries J. D. . Vinculin controls focal adhesion formation by direct interactions with talin and actin. J. Cell Biol. 179, 1043–1057 (2007). 18056416 10.1083/jcb.200703036 PMC2099183

[b45] Lee H. S., Lim C. J., Puzon-McLaughlin W., Shattil S. J. & Ginsberg M. H. RIAM activates integrins by linking talin to ras GTPase membrane-targeting sequences. J. Biol. Chem. 284, 5119–5127 (2009). 19098287 10.1074/jbc.M807117200 PMC2643525

[b46] Wang R. S., Saadatpour A. & Albert R. Boolean modeling in systems biology: an overview of methodology and applications. Phys. Biol. 9, 055001 (2012). 23011283 10.1088/1478-3975/9/5/055001

[b47] Efimov A. . Paxillin-dependent stimulation of microtubule catastrophes at focal adhesion sites. J. Cell Sci. 121, 196–204 (2008). 18187451 10.1242/jcs.012666 PMC3164837

[b48] Krylyshkina O. . Modulation of substrate adhesion dynamics via microtubule targeting requires kinesin-1. J. Cell Biol. 156, 349–359 (2002). 11807097 10.1083/jcb.200105051 PMC2199234

[b49] Birukova A. A. . Microtubule disassembly induces cytoskeletal remodeling and lung vascular barrier dysfunction: role of Rho-dependent mechanisms. J. Cell Physiol. 201, 55–70 (2004). 15281089 10.1002/jcp.20055

[b50] Smilenov L., Forsberg E., Zeligman I., Sparrman M. & Johansson S. Separation of fibronectin from a plasma gelatinase using immobilized metal affinity chromatography. FEBS Lett. 302, 227–230 (1992). 1318226 10.1016/0014-5793(92)80447-o

[b51] Tomasi C. & Manduchi R. Bilateral filtering for gray and color images. IEEE Comp. Soc. XVIII, 1164S (1998).

[b52] Lock J. G. & Stromblad S. Systems microscopy: an emerging strategy for the life sciences. Exp. Cell Res. 316, 1438–1444 (2010). 20381488 10.1016/j.yexcr.2010.04.001

