# Supplementary material for: A plastic relationship between vinculin-mediated tension and adhesion complex area defines adhesion size and lifetime
Source: Nat Commun. 2015 Jun 25;6:7524. doi: 10.1038/ncomms8524 (PMC4491829; doi:10.1038/ncomms8524)
Supplement: Supplementary Information — Supplementary Figures 1-8 [file ncomms8524-s1.pdf]

SF1

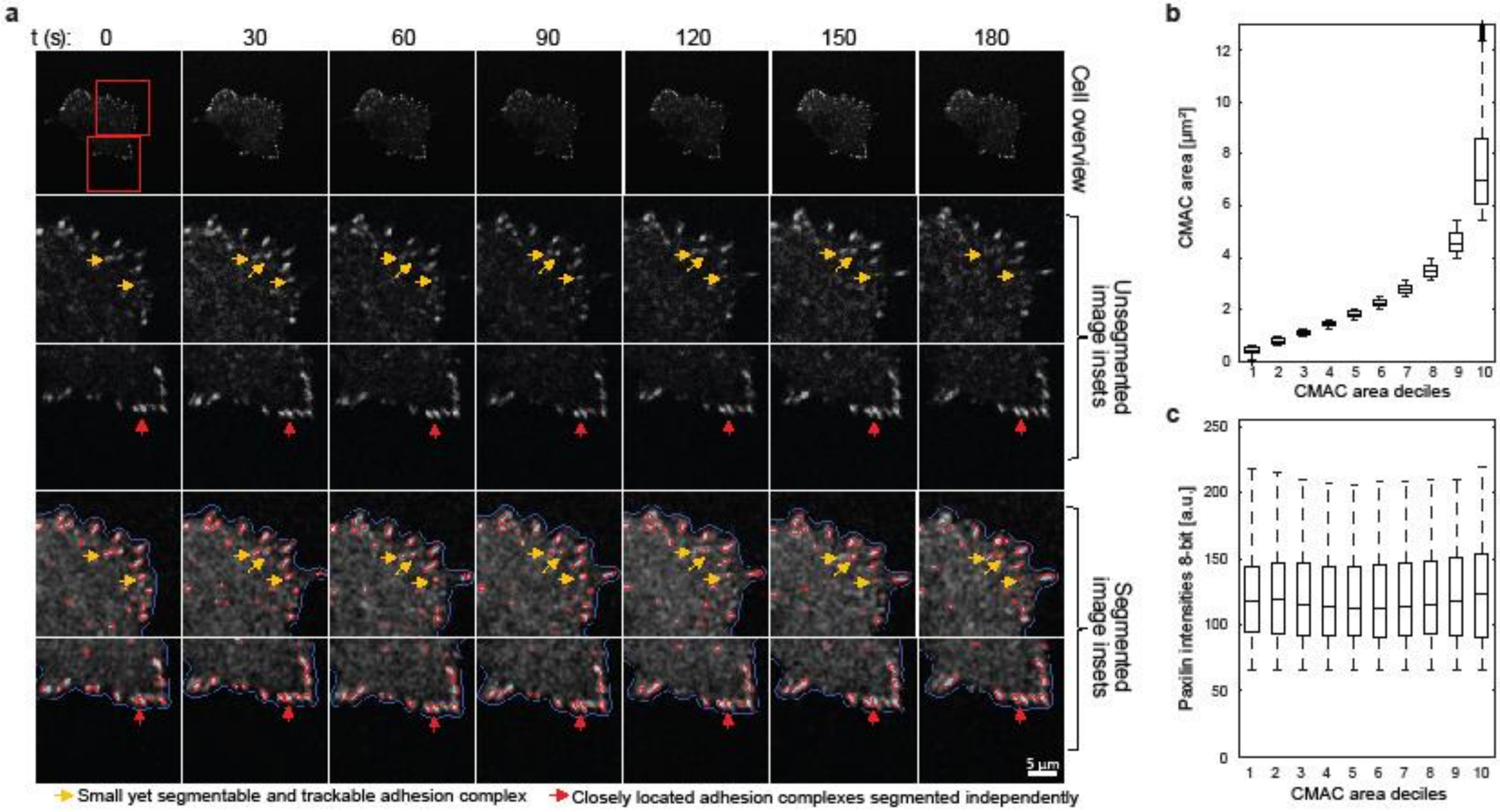
a
t (s): 0 30 60 90 120 150 180
Cell overview
Unsegmented image insets
Segmented image insets
5 µm
Small yet segmentable and trackable adhesion complex
Closely located adhesion complexes segmented independently
b
CMAC area [µm²]
CMAC area deciles
c
Paxilin intensities 8-bit [a.u.]
CMAC area deciles

Supplementary Figure 1:

Segmentation and fluorescent intensity quantification

a) Representative image time series showing segmentation accuracy over a period of 180 s. The top row depicts a whole cell from which two inserts are extracted (red squares). These two inserts are magnified in panel rows two to five (from top) with both the unsegmented (rows two and three) and the segmented cell adhesions (rows four and five). Red arrows denote two proximal adhesions that nevertheless segmented accurately as separate objects; yellow arrows highlight small, yet robustly segmented and tracked adhesions. NB: Segmented objects that are only present at single time points (cf. row four) are excluded from further analysis, as each object has to be tracked in minimally 4 consecutive time points. This ensures that neither shot noise nor cytoplasmic signal contribute to quantitative analyses. Bar 5 µm.

b) CMAC area distributions and c) paxilin intensities of CMACs for each CMAC area decile represented as boxplots (n=2919 CMACs). No differences are observed in paxilin intensity, indicating that CMAC size does not bias intensity values and therefore does not imply biasing of V-tension values.

SF2

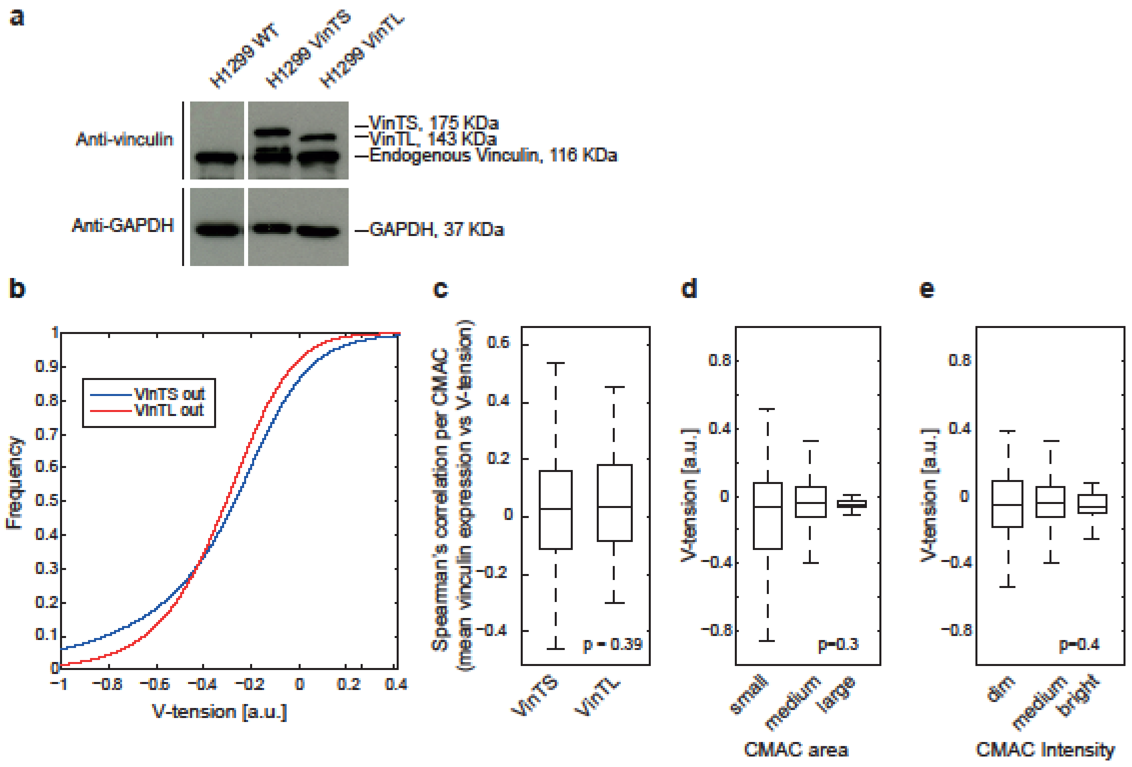

Supplementary Figure 2:

VinTS tension sensor controls

a) Immunoblot of vinculin expression levels comparing endogeneous vinculin expression to the stable expression of the VinTS and VinTL constructs. Both constructs were expressed at lower levels than the endogeneous vinculin expression level. Sizes of different vinculin species are indicated. Blotting for GAPDH was used as a loading control.

b) cell-matrix adhesion complex (CMAC) segmentation masks were repeatedly randomly repositioned within cells (excluding CMAC regions) and V-tension values calculated. This provides a comparable sampling of V-tension signals in cytoplasmic regions. Values from VinTS and VinTL constructs are plotted as cumulative distribution functions (CDFs). Only very small differences in signal between VinTS and VinTL are observed outside CMACs. Given that no vinculin-transmitted tension is expected outside CMACs, this supports the tension-dependence of the VinTS signals detected within CMACs (Figure 2).

c) Median Spearman's correlations between sensor-concentration (intensity) and V-tension signal within CMACs do not differ between VinTS and VinTL (p value based on Kruskal-Wallis test).

d) Medians of vinculin-mediated tension (V-tension)-distributions from very small (0.1%ile: <0.2 μm^2) and very large (99.9%ile: >17.1 μm^2) CMACs do not differ from those of the remaining cell adhesion complexes (p value based on Kruskal-Wallis-test with Tukey-Kramer multiple comparison adjustment).

e) Medians of V-tension-distributions from very dim (0.1%ile: <19 8-bit intensity units) and very bright (99.9%ile: >123 8-bit intensity units) CMACs do not differ from those of the remaining cell adhesion complexes (p value based on Kruskal-Wallis-test with Tukey-Kramer multiple comparison adjustment).

SF3

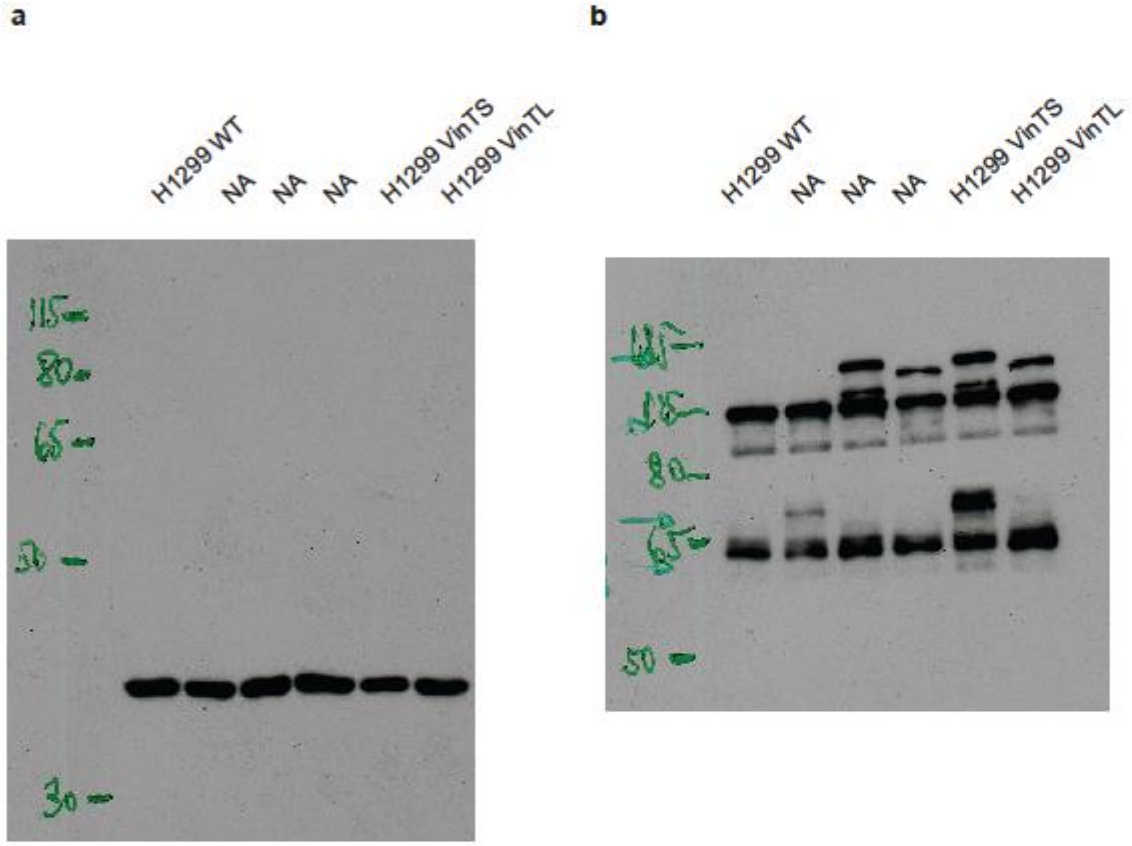

Supplementary Figure 3:

Scans of films of immunoblotting assays shown in Supplementary Figure 2a

Raw scans of immunoblot films for data shown in Supplementary Figure 2a. The membrane was cut into three pieces before blotting (middle piece not used in this study). Displayed are blotting for Vinculin (top) and loading control GAPDH (bottom). NA refers to cells not used in this study.

SF4

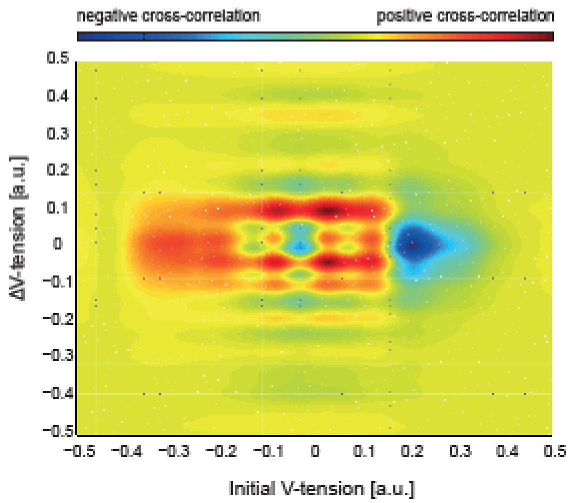

Supplementary Figure 4:

Vinculin-mediated tension-conditioned cross-correlation map

Vinculin-mediated tension (V-tension) -conditioned cross-correlation map showing the net probability of positive (red) or negative (blue) cross-correlation between V-tension and cell-matrix adhesion complex (CMAC) area in the V-tension/ΔV-tension space. X-axis: initial V-tension (value T_0 in Figure 3b). Y-axis: change in V-tension (calculated as T_1-T_0 in Figure 2B). Red regions indicate where positive cross-correlation is more probable; blue regions indicate where negative cross-correlation is more probable. 768 CMACs from 25 VinTS expressing cells passed the confidence criterion, in contrast to only 72 CMACs in VinTL expressing cells.

SF5

a **VinTL**

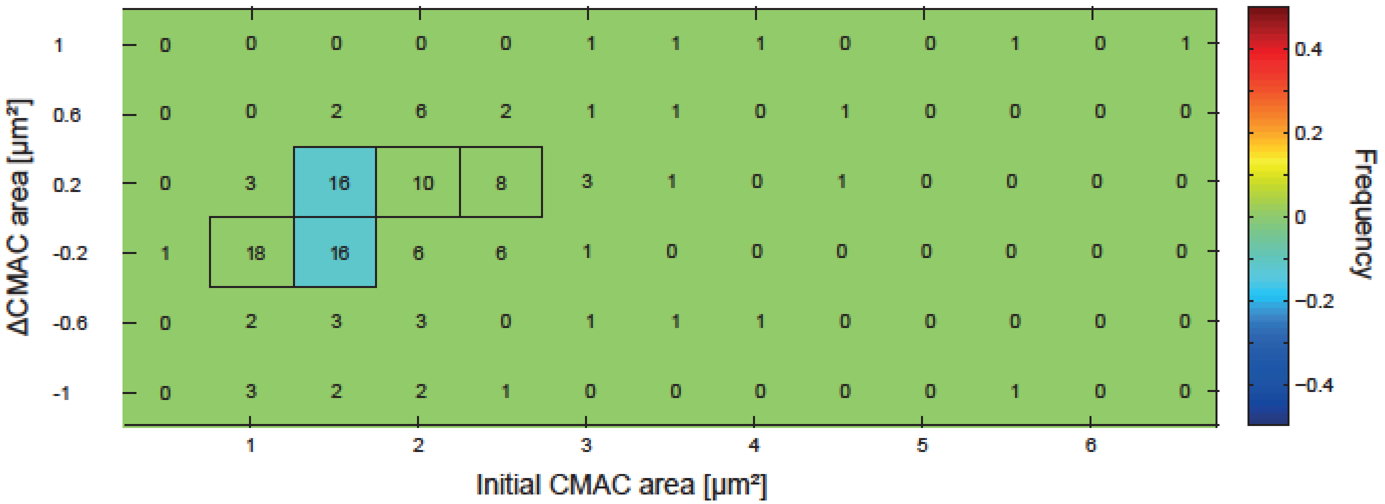

b **VinTS**

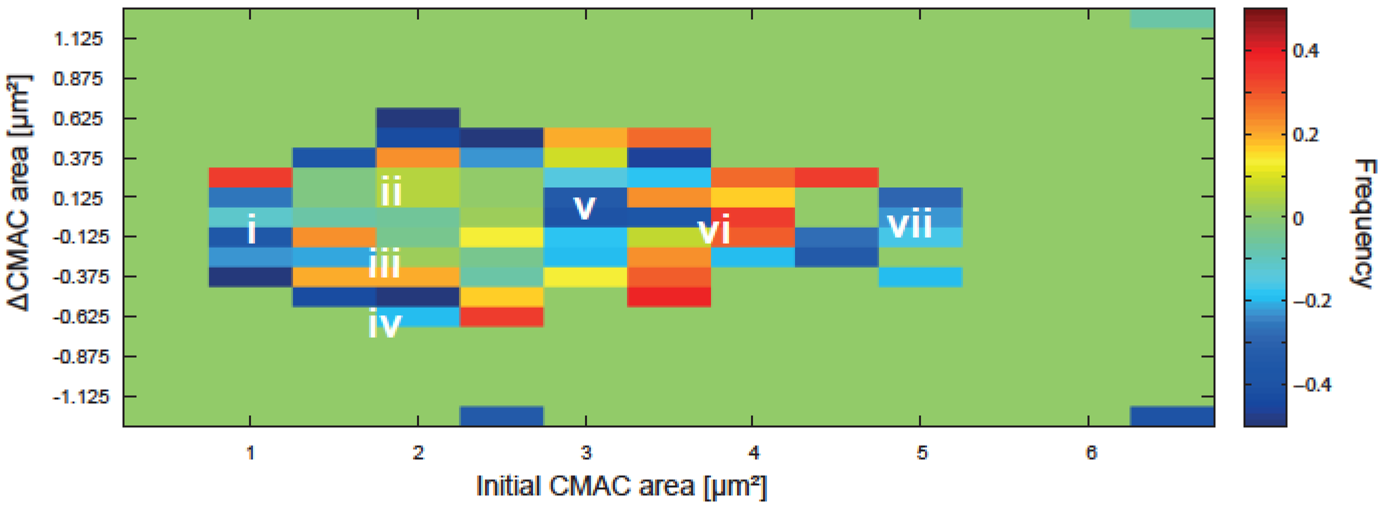

Supplementary Figure 5:

Area-conditioned cross-correlation maps as 3D-histograms

X-axis denotes initial CMAC area; Y-axis denotes the change in CMAC area. Due to the low number of significant cross-correlations observed in the VinTL dataset, a Kernel-density estimation was not suitable. Therefore the Initial CMAC area/ΔCMAC area space was binned and the frequency of positive (red) or negative (blue) cross-correlation calculated. A minimal number of 8 observations per bin was chosen to be representative (black frames in a) accounting for an error-rate of maximally 0.125.

a) Colour-coded 3D-histogram of cross-correlation frequencies in the VinTL dataset. Numbers in each bin indicate the observation counts. The VinTL map supports neither a strong nor a structured context-dependence.

b) Colour-coded 3D-histogram of cross-correlation frequencies in the VinTS dataset. Regions i to iv correspond to those in Figure 4. Region v is represented in Figure 3A as the blue area between 2 and 3 μm^2; region vi between 3 and 4 μm^2 and region vii between 5 and 6 μm^2.

SF6

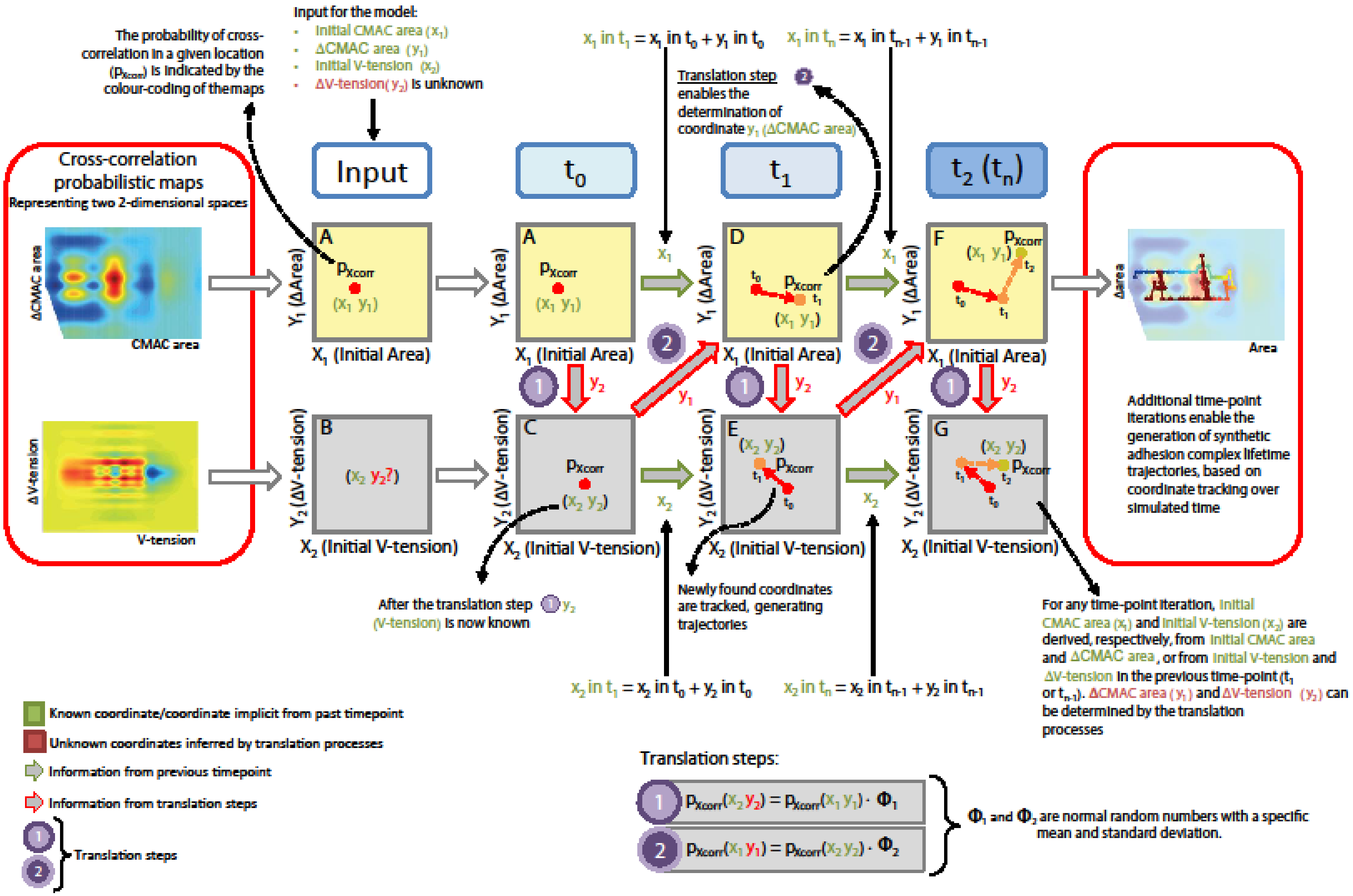

The probability of cross-correlation in a given location (PXcorr) is indicated by the colour-coding of the maps
Input for the model:
Initial CMAC area (x1)
ΔCMAC area (y1)
Initial V-tension (x2)
ΔV-tension (y2) is unknown
x1 in t1 = x1 in t0 + y1 in t0
x1 in tn = x1 in tn-1 + y1 in tn-1
Translation step 2 enables the determination of coordinate y1 (ΔCMAC area)
Cross-correlation probabilistic maps
Representing two 2-dimensional spaces
ΔCMAC area
CMAC area
Δ V-tension
V-tension
Input
t0
t1
t2 (tn)
A
D
F
B
C
E
G
PXcorr
(x1 y1)
(x2 y2?)
(x2 y2)
Y1 (ΔArea)
X1 (Initial Area)
Y2 (ΔV-tension)
X2 (Initial V-tension)
x1
x2
y1
y2
t0
t1
t2
Δarea
Area
Additional time-point iterations enable the generation of synthetic adhesion complex lifetime trajectories, based on coordinate tracking over simulated time
After the translation step 1 y2 (V-tension) is now known
Newly found coordinates are tracked, generating trajectories
x2 in t1 = x2 in t0 + y2 in t0
x2 in tn = x2 in tn-1 + y2 in tn-1
For any time-point iteration, Initial CMAC area (x1) and Initial V-tension (x2) are derived, respectively, from Initial CMAC area and ΔCMAC area, or from Initial V-tension and ΔV-tension in the previous time-point (t1 or tn-1). ΔCMAC area (y1) and ΔV-tension (y2) can be determined by the translation processes
Known coordinate/coordinate implicit from past timepoint
Unknown coordinates inferred by translation processes
Information from previous timepoint
Information from translation steps
Translation steps
Translation steps:
1 PXcorr(x2 y2) = PXcorr(x1 y1) · Φ1
2 PXcorr(x1 y1) = PXcorr(x2 y2) · Φ2
Φ1 and Φ2 are normal random numbers with a specific mean and standard deviation.

Supplementary Figure 6:

Schematic description of the modelling process

Single cell-matrix adhesion complex (CMAC) trajectories were modelled using the area/Δarea and the V-tension/ΔV-tension cross-correlation probability maps (left red box). Briefly, we determined synthetic CMAC coordinates within the area/Δarea and within the V-tension/ΔV-tension probability maps for each of the iterated time-points. The locations are alternated between these maps per time point, and are then tracked over simulated time to generate synthetic adhesion complex trajectories.

The inputs for the models are the initial area (CMAC area at t_0, x_1), the initial growth (ΔCMAC area at t_0, y_1) and the median initial V-tension (V-tension at t_0, x_2), simulating a small adhesion that is growing (as defined in Methods). The initial area and the initial growth define coordinates within the CMAC area/ΔCMAC area map, which have an associated probability of positive or negative cross-correlation (***pXcorr***).

While the initial V-tension is an input for the model, ΔV-tension (y_2) for the initial time-point is unknown, and has to be determined in order to complete the corresponding coordinates on the V-tension/ΔV-tension map. This missing variable can be estimated using the probability of cross-correlation associated with the CMAC area/ΔCMAC area coordinates at t_0 modified by a stochastic translation factor $\boldsymbol{\Phi_1}$ (a normal random number with specified mean and standard deviation).

For instance, given a small CMAC (Initial CMAC area = 0.2 μm²) that is growing (ΔCMAC Area = 0.1 μm²) with an initial V-tension of 0.1 a.u., the type of correlation is known: A medium negative cross-correlation probability means that the V-tension value at the next iteration should be smaller than 0.1 a.u. Hence, this cross-correlation value is multiplied with a normal random number $\boldsymbol{\Phi_1}$ to estimate the V-tension value at time t_1. Each model is characterised by co-varying the mean and/or standard deviation of the translation factor $\boldsymbol{\Phi_1}$.

This translation process (1, indicated by the red arrow) renders a new ΔV-tension coordinate (y_2), defining a localisation within the V-tension/ΔV-tension space at t_0. This localisation is associated with a new probability of cross-correlation.

Once all coordinates for t_0 are known, the first time iteration can be performed. The synthetic CMAC area at t_1 can be easily calculated as the sum of CMAC area (x_1) and ΔCMAC area (y_1) in the *previous* time-point (t_0). However, ΔCMAC area (y_1) for the *current* time-point is unknown, and has to be determined in order to locate the coordinates on the CMAC area/ΔCMAC area map. A new translation process (2, also indicated by red arrows) renders the missing coordinate, directed by the associated probability of cross-correlation in the V-tension/ΔV-tension map at t_0 modified by a stochastic translation factor $\boldsymbol{\Phi_2}$,. Thus, a new localisation within the CMAC area/ΔCMAC area map can be determined. Comparing the current localisation to the previous one, the trajectory of the simulated object in the CMAC area/ΔCMAC area space can be observed.

Although V-tension at the current time iteration (t_1) can be calculated from summing *previous* V-tension (x_2) and ΔV-tension data (y_2) at t_0, *current* ΔV-tension is yet to be determined. Similarly to the determination of ΔV-tension for t_0, a translation process driven by the stochastic factor $\boldsymbol{\Phi_1}$ and the probability of cross-correlation associated to the newly found location on the CMAC area/ΔCMAC area map enables the determination of the missing variable for the current time-point. Given the current and past coordinates in the V-tension/ΔV-tension map, tracking can also be done in this space.

For any time-point iteration (t_n), CMAC area (x_1) and V-tension (x_2) are easily derived, respectively, from summing CMAC area and ΔCMAC area ($x_1 + y_1$), or from summing V-tension and ΔV-tension ($x_2 + y_2$), in the *previous* time-point (t_{n-1}). However, ΔCMAC area (y_1) and ΔV-tension (y_2) for the *current* time-point t_n have to be determined. Again, this is possible using the translation processes directed by the probability of cross-correlation associated with the previously determined coordinates, modified by the stochastic components $\boldsymbol{\Phi_1}$ and $\boldsymbol{\Phi_2}$. The new coordinates can be then located on their respective map. Tracks marking the localisations found for all time-points build up individual adhesion complex trajectories in these spaces (as exemplified in the CMAC area/ΔCMAC area probability map, red box, right).

SF7

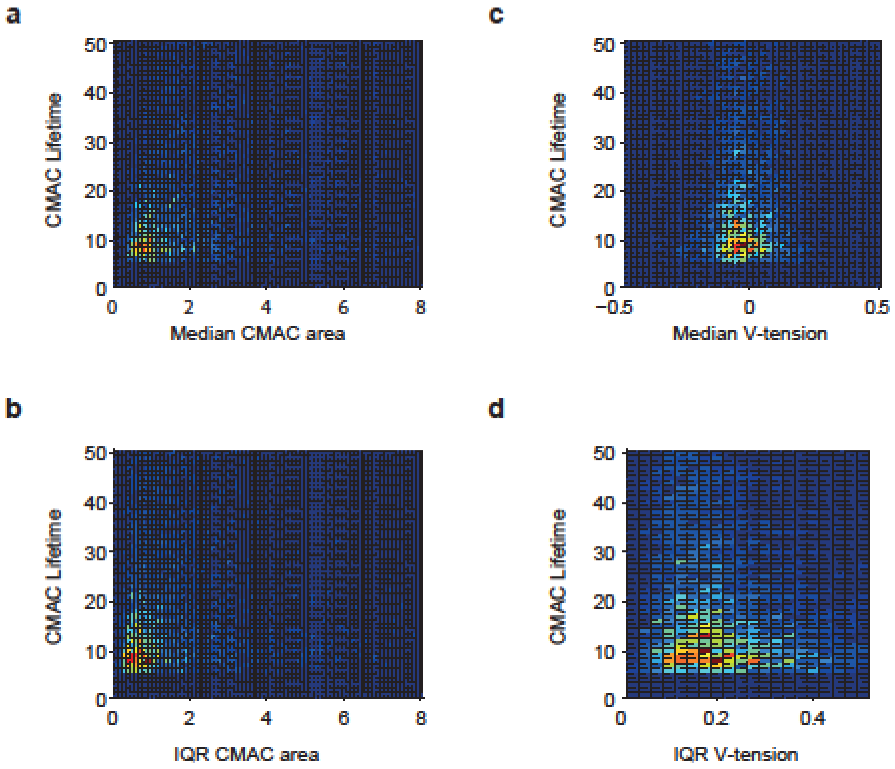

Supplementary Figure 7:

Adhesion complex lifetime independent of model input variables

Cell-matrix adhesion complex (CMAC) lifetime, CMAC area and vinculin-mediated tension (V-tension) features do not show dependence. Heat-maps of CMAC Lifetime and median CMAC area (a), inter-quartile range (IQR) of CMAC area (b), median V-tension (c), and IQR of V-tension (d) are shown. The lack of a clear pattern along the diagonal of the plots indicates the independence of the assessed variables.

SF8

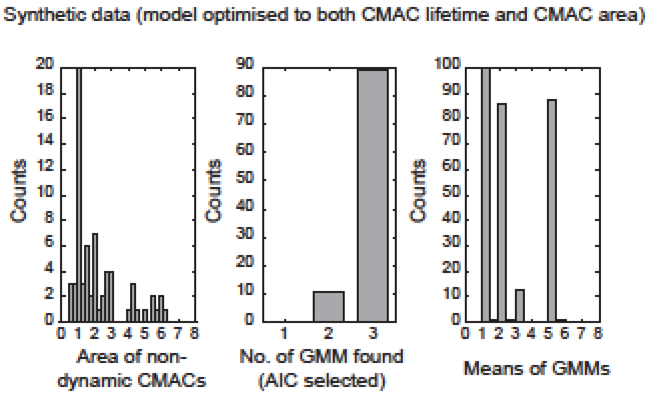

Supplementary Figure 8:

Metastable subpopulations predicted by an alternative model

Replication of analysis in Figure 5C with synthetic data from an alternative model optimized to both cell-matrix adhesion complex (CMAC) lifetime and CMAC area value prediction. The CMAC area distribution of synthetic non-dynamic CMACs (Figure 6b, from model optimized to both CMAC lifetime and CMAC area) shows a non-random distribution (left panel). Akaike information criterion (AIC)-based selection of Gaussian mixture models (GMMs) suggests the presence of 2 or 3 subpopulations (centre panel). The means of GMM-predicted subpopulations cluster around CMAC area values of ~1-3, and ~5-6 μm², suggesting the existence of

underlying stable steady-states (right panel). Subpopulations means are similar to those predicted by the model in Figure 6c and observed in empirical data (Figure 6d).
